# Integrity of the LXXLL motif in Stat6 is required for the inhibition of breast cancer cell growth and enhancement of differentiation in the context of progesterone

**DOI:** 10.1186/1471-2407-14-10

**Published:** 2014-01-08

**Authors:** Min Wei, Qi He, Zhongyin Yang, Zhiwei Wang, Qing Zhang, Bingya Liu, Qinlong Gu, Liping Su, Yingyan Yu, Zhenggang Zhu, Guofeng Zhang

**Affiliations:** 1 Breast Department, International Peace Maternity and Child Health Hospital, Shanghai Jiaotong University, Shanghai 200030, People’s Republic of China; 2 Key Laboratory of Shanghai Gastric Neoplasms, Department of Surgery, Shanghai Institute of Digestive Surgery, Ruijin Hospital, School of Medicine, Shanghai Jiao Tong University, Shanghai 200025, People’s Republic of China; 3 Department of General Surgery, Tongji Hospital, Tongji University School of Medicine, Shanghai 200025, People’s Republic of China

**Keywords:** Breast cancer, Stat6, p21, p27

## Abstract

**Background:**

Progesterone is essential for the proliferation and differentiation of mammary gland epithelium. Studies of breast cancer cells have demonstrated a biphasic progesterone response consisting of an initial proliferative burst followed by sustained growth arrest. However, the transcriptional factors acting with the progesterone receptor (PR) to mediate the effects of progesterone on mammary cell growth and differentiation remain to be determined. Recently, it was demonstrated that signal transducer and activator of transcription 6 (Stat6) is a cell growth suppressor. Similar to progesterone-bound PR, Stat6 acts by inducing the expression of the G1 cyclin-dependent kinase inhibitors p21 and p27. The possible interaction between Stat6 and progesterone pathways in mammary cells was therefore investigated in the present study.

**Methods:**

ChIP and luciferase were assayed to determine whether Stat6 induces p21 and p27 expression by recruitment at the proximal Sp1-binding sites of the gene promoters. Immunoprecipitation and Western blotting were performed to investigate the interaction between Stat6 and PR-B. The cellular DNA content and cell cycle distribution in breast cancer cells were analyzed by FACS.

**Results:**

We found that Stat6 interacts with progesterone-activated PR in T47D cells. Stat6 synergizes with progesterone-bound PR to transactivate the p21 and p27 gene promoters at the proximal Sp1-binding sites. Moreover, Stat6 overexpression and knockdown, respectively, increased or prevented the induction of p21 and p27 gene expression by progesterone. Stat6 knockdown also abolished the inhibitory effects of progesterone on pRB phosphorylation, G1/S cell cycle progression, and cell proliferation. In addition, knockdown of Stat6 expression prevented the induction of breast cell differentiation markers, previously identified as progesterone target genes. Finally, Stat6 gene expression levels increased following progesterone treatment, indicating a positive auto-regulatory loop between PR and Stat6.

**Conclusions:**

Taken together, these data identify Stat6 as a coactivator of PR mediating the growth-inhibitory and differentiation effects of progesterone on breast cancer cells.

## Background

The steroid hormones estrogen and progesterone play key roles in the growth of the mammary gland [1]. Estrogens appear to be the main drivers of proliferation of the mammary gland epithelium, whereas progesterone is required for its terminal growth and differentiation [2]. The induction of mammary epithelial development during pregnancy is mediated by a rise in progesterone levels [3,4]. Progesterone exerts its physiological effects mainly via interaction with specific intracellular progesterone receptors (PRs), PR-A and PR-B, which are products of a single gene and are members of the nuclear receptor (NR) family [5]. Studies on mice in which the expression of both PRs was ablated have demonstrated that progesterone is necessary for ductal branching and the lobulo-alveolar development of the mammary gland [6]. More recently, selective ablation of each receptor isoform has indicated that PR-B is specifically required for the progesterone-dependent development of the mammary gland during pregnancy [7].

In relation to the function of progesterone in breast development, both growth-stimulatory and -inhibitory effects on breast epithelium cells and cancer development have been reported in animal tumor models [8-10]. Moreover, in vitro studies using the PR-positive mammary carcinoma T47D cell line as a model have demonstrated a biphasic cellular response to either progesterone or its derivatives (R5020 or ORG2058), with an immediate proliferative burst followed by a sustained growth arrest [11-13]. As with many hormones and growth factors, the regulation of retinoblastoma gene product (pRB) phosphorylation, a critical checkpoint in the G1/S transition, plays a major role in the control of proliferation by progesterone [14-16]. The initial pRB phosphorylation provoked by progesterone is catalyzed by constitutively-expressed cyclin-dependent kinases (CDKs), which are activated through interaction with specific cyclins induced by progesterone [14,15,17]. The ensuing growth arrest is associated, at least in part, with the transitory induction of cyclin-dependent kinase inhibitors (CDKIs) p21 and p18, followed by sustained induction of p27 [18-20]. Associations of these CDKIs with the different G1 CDK complexes led to inhibition of their activity and a decrease in pRB phosphorylation, resulting in cell cycle arrest in late G1 phase. It is known that progesterone induces the expression of both p21 and p27 through a transcriptional mechanism that involves interaction between progesterone-bound PR, the general coactivator CBP/p300, and the transcription factor Sp1 at proximal Sp1-binding sites [19,21]. However, since PR is expressed during both phases of the progesterone response [11-14], unidentified PR target genes and/or cofactors of PR are likely to be involved with it in the delayed growth-inhibitory effects of progesterone.

Signal transducer and activator of transcription 6 (Stat6) was isolated as a novel factor implicated in the regulation of various cytokine genes [22,23]. Recently, we identified a new function for Stat6 as a growth suppressor protein in CHO and mammary cancer cells (in submission). As with PR, the antiproliferative activity of Stat6 involves its interaction with Sp1 to activate the p21 and p27 promoters, resulting in the inhibition of G1 CDK-mediated phosphorylation of pRB and histone H1. In view of the ability of Stat6 to function as a nuclear receptor coactivator, in this study we tested whether Stat6 interacts with PR and influences the progesterone-dependent regulation of mammary cancer cell growth. Using the T47D cell line as a model, we show that Stat6 is indeed a coactivator of PR at the p21 and p27 gene promoters. Furthermore, we show that Stat6 gene expression itself is steadily induced by progesterone, which is necessary for the long-term growth-inhibitory and differentiating effects of the hormone. Thus, Stat6 is likely to mediate a positive feedback loop in the progesterone response that is crucial for the delayed and sustained action of progesterone on breast cancer cells.

## Methods

### Plasmids

The Stat6 expression vector subcloned in pCMV4-flag was constructed as previously described. The 2.4-kilobase pair genomic fragment containing the transcription start site of P21 was subcloned into the HindII site of the luciferase reporter vector, pGL3-Basic (Promega), to generate P21Luc. The p27 promoter reporter constructs were a gift from Dr. Toshiyuki Sakai (Kyoto Prefectural University of Medicine) [24].

### Cell culture assays

Human T47D ductal carcinoma cells, a model commonly used to study progesterone signaling in breast cancer cells, were obtained from the American Type Culture Collection (Rockville, MD) and were cultured as a monolayer as previously described [25]. In all assays, the cells were first synchronized in G0/G1 phase by a double thymidine block as previously described [26]. Progesterone (30 nM) or ethanol (vehicle) was added daily when the cells resumed proliferation by reincubation in routine growth medium (corresponding to time zero of the experiments). Each experiment was repeated at least three times, and the results are presented as means ± standard deviations (SD) of a representative experiment carried out in triplicate. Cellular DNA content and flow cytometry profiles were determined, respectively, by the staining of nuclear DNA using the fluorochrome 3,5-diaminobenzoic acid (free acid) and propidium iodide as described previously [27]. Transient transfections were performed with Lipo2000 (Invitrogen, Carlsbad, USA) as previously described [28]. Chromatin immunoprecipitation assays were performed as described elsewhere [28] using 40 μg of anti-Stat6 (Abcam), anti-Progesterone Receptor, (anti-PR, specific for the β-form of PR) (Abcam), and anti-p300 (Santa Cruz) and anti-Sp1 (Santa Cruz) antibodies for the immunoprecipitation of cell lysates. Briefly, T47D cells were subjected to chromatin immunoprecipitation (ChIP) with the ChIP Assay kit (Upstate Cell Signaling Solutions). Briefly, cross-linking of proteins with DNA was done with 4% formaldehyde at 37°C for 15 minutes and quenched with glycine. Cell lysates were sonicated (Branson Sonifier) to shear the DNA to 400- to 1,000-bp length fragments. Chromatin samples were then precleared with a salmon sperm DNA/protein A agarose 50% slurry for 30 minutes at 4°C and immunoprecipitated overnight in the absence of antibody or with antibodies for flag, Stat6, PR, Sp-1, and p300. The PCR products were separated on a 2% agarose gel, stained with ethidium bromide, and visualized under UV light.

### Immunostaining

T47D cells, subconfluently grown on glass coverslips, were transfected with small interfering RNA (siRNA), treated with progesterone or ethanol (vehicle) for 48 h, and then fixed and permeabilized with 4% formaldehyde and 0.5% Triton X-100 in PBS for 10 minutes. For fluorescent immunocytochemistry, the cells were first permeabilized by boiling in 10 mM citrate buffer. The rabbit polyclonal Stat6 antibody (1:50; Abcam) was then detected with an FITC-conjugated goat anti-rabbit immunoglobulin G (1:500; Sigma). Following three washes with PBS, the cells were incubated with an actin-specific marker, phalloidin (Sigma). After three washes, the coverslips and their attached cells were mounted on glass microscope slides using mounting medium with DAPI (Molecular Probes). To detect lipid, cells were stained with Oil Red O and counterstained with hematoxylin. Specimens were visualized and photographed using a Leica TCS-SP2 confocal microscope (for fluorescent immunocytochemistry) or a Leica DC480 color video camera (for Oil Red O staining). Oil Red O staining intensity was quantified as described in [29]. Results represent the means ± SD of values from a single experiment (nΧ6fields/point) repeated three times with similar results.

### Reverse transcription and quantitative PCR

Transcript levels in extracted total RNA were assessed by quantitative reverse transcription-PCR (RT-PCR) using the oligonucleotide primers specific for human Stat6, p21, and p27 as described previously (24). In addition, the following primer pairs were used: desmoplakin, 5′-TGATAAACTCAGACAGCGCC-3′ and 5′-CATCAAACACCAGCTTGGAG-3′; Na/K-ATPase-α1, 5′-CTGGCTTGAGGCTGTCATCTTCCTC-3′ and 5′-TTCCTTGCCATGCGTTTGGC-3′; fatty acid synthase (FAS), 5′-ATCGTGGACGGAGGCATCAACC-3′ and 5′-TTGGCCATCATCGCTCGCTG-3′; non-tissue-specific alkaline phosphatase (ALP), 5′-TCACTCTCCGAGATGGTGGTGGTGG-3′ and 5′-TTCCTTCATGGTGCCCGTGG-3′. Because of their stability during cell cycle progression, GADPH levels were simultaneously quantified for normalization. Each figure indicates mRNA levels as means ± SD (n = 3).

### Knockdown of Stat6 expression

Stat6 expression was knocked-down using siRNA as described elsewhere [30]. Briefly, the oligonucleotides used to generate three Stat6 siRNAs targeting three distinct regions of Stat6 cDNA (siRNA-1: 5′-GGGAGAAGAUGUGUGAAACUCUGAA-3′, siRNA-2: 5′-GAAUCCGGGAUCUUGCUCAGCUCAA-3′, and siRNA-3:5′-CAGUUCCGCCACUUGCCAAdTdT-3′) were synthesized by Invitrogen. The nonsilencing siRNA oligonucleotide, which does not target any known mammalian gene and is used as a negative control, was from Ambion. siRNA duplexes (500 ng) were transfected at 0 and 3 days using Lipo2000 (Invitrogen, Carlsbad, USA). Down-regulation of the target gene (Stat6) by specific siRNA but not by negative controls was confirmed by Western blotting (Additional file 1: Figure S1). Representative experiments have been performed with Stat6 siRNA-3.

### Protein assays

Immunoprecipitation assays were performed as previously described [31]. Cells were washed twice with PBS, collected and homogenized with RIPA buffer. After cell debris was removed by centrifugation, extracts were aliquoted and either used immediately or stored at -80°C. Whole-cell lysates in lysis buffer were cleared with 1.0 μg nonimmune rabbit IgG (Santa Cruz) together with 30 μl of protein A-Sepharose beads (Pierce). After centrifugation, the lysates were immunoprecipitated for 1 h at 4°C with 1 μg of the anti-Stat6 antibody or nonimmune rabbit IgG and then incubated overnight at 4°C with protein A-Sepharose. The immunoprecipitates were washed three times with lysis buffer and once with PBS and then resuspended in electrophoresis sample buffer. Samples of immunoprecipitated or total proteins (30 μg) were analyzed by Western blotting using the anti-ppRB-Ser807/811 antibody (Cell Signaling Technology) against a pRB peptide phosphorylated on the Ser807/811 residue, which is phosphorylated by both CDK2 and CDK4/6 kinases [32], or the anti-pRB against underphosphorylated pRB (BD Biosciences-Pharmingen), the anti-PR antibody (abcam), anti-p21(abcam), anti-p27(abcam), and anti-GADPH (as control antibody). The blots represent typical results from at least three independent experiments.

### Statistical analyses

Statistical analyses were performed using the nonparametric Mann–Whitney test.

## Results

### Stat6 enhances the progesterone response of the p21 and p27 gene promoters

**Figure 1:**
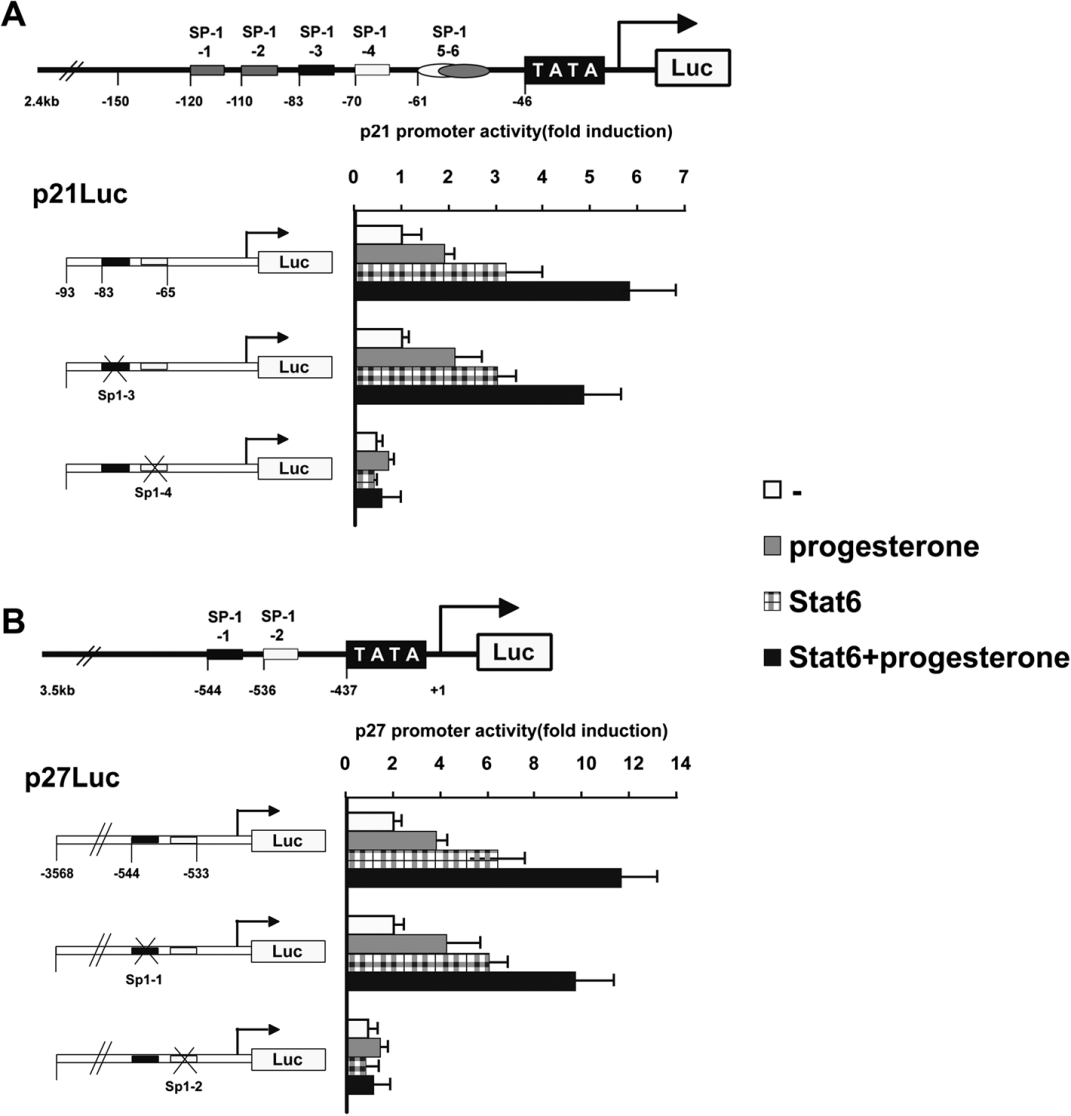
**Stat6 enhances p21 and p27 promoter activities induced by progesterone.** T47D cells were transiently cotransfected with the reporter constructs containing the indicated p21 **(A)** or p27 **(B)** promoter fragments. Twelve hours after transfection, cells were incubated with progesterone (30 nM) or vehicle (ethanol) for 24 h and then harvested for the luciferase activity assay. Results are expressed as increase (mean ± SD) over luciferase activity levels in control (-) p21Luc or p27Luc, arbitrarily set as 1. The arrows represent the transcription start sites; the crosses indicate the mutated Sp1-binding sites. For each promoter construct, columns followed by different symbols are statistically significantly different from each other.

Previously, we demonstrated that Stat6 induced the p21 and p27 genes by interacting with Sp1 through the proximal Sp1-binding elements (comprising the Sp1-3 and Sp1-4 sites for p21 and the Sp1-1 and Sp1-2 sites for p27). Coincidentally, progesterone-bound PR has been shown to activate the p21 and p27 genes by interacting with Sp1 through the same proximal Sp1-binding elements [19,21]. Therefore, it was hypothesized that Stat6 and PR could interact functionally at these proximal Sp1 response elements to activate transcription of both promoters. To test this, wild type p21 or p27 promoter reporter constructs (denoted p21Luc and p27Luc, respectively) were cotransfected with the Stat6 expression plasmid in PR-positive breast carcinoma T47D cells [33,34], and the cells were treated with progesterone or left untreated (Figure 1). Confirming the results of previous studies [19,21], Stat6 or progesterone treatment alone stimulated both p21 and p27 gene promoter activities. Interestingly, a synergistic effect of Stat6 and progesterone was observed on both CDKI promoters. To assess the roles of the Sp1 sites in this response further, the p21 and p27 reporter constructs mutated at each Sp1 site were transiently cotransfected with Stat6 in cells either untreated or incubated with progesterone (Figure 1). As previously reported, the mutation of the Sp1-3 or the Sp1-1 sites diminished the basal activity and abolished the responses of the p21 or p27 promoters to Stat6 or progesterone alone [19,21,35]. Moreover, mutation of the Sp1-4 or the Sp1-2 site reduced the progesterone-dependent transactivation of the p21 or p27 promoter, respectively. However, mutation of each of these sites prevented the synergistic effects of Stat6 and progesterone on both promoters. These results indicate that Stat6 cooperates with the progesterone pathway to transactivate the proximal Sp1 response elements of the p21 and p27 gene promoters.

### Stat6 is recruited by progesterone-activated PR at the proximal Sp1-binding sites of the p21 and p27 gene promoters

**Figure 2:**
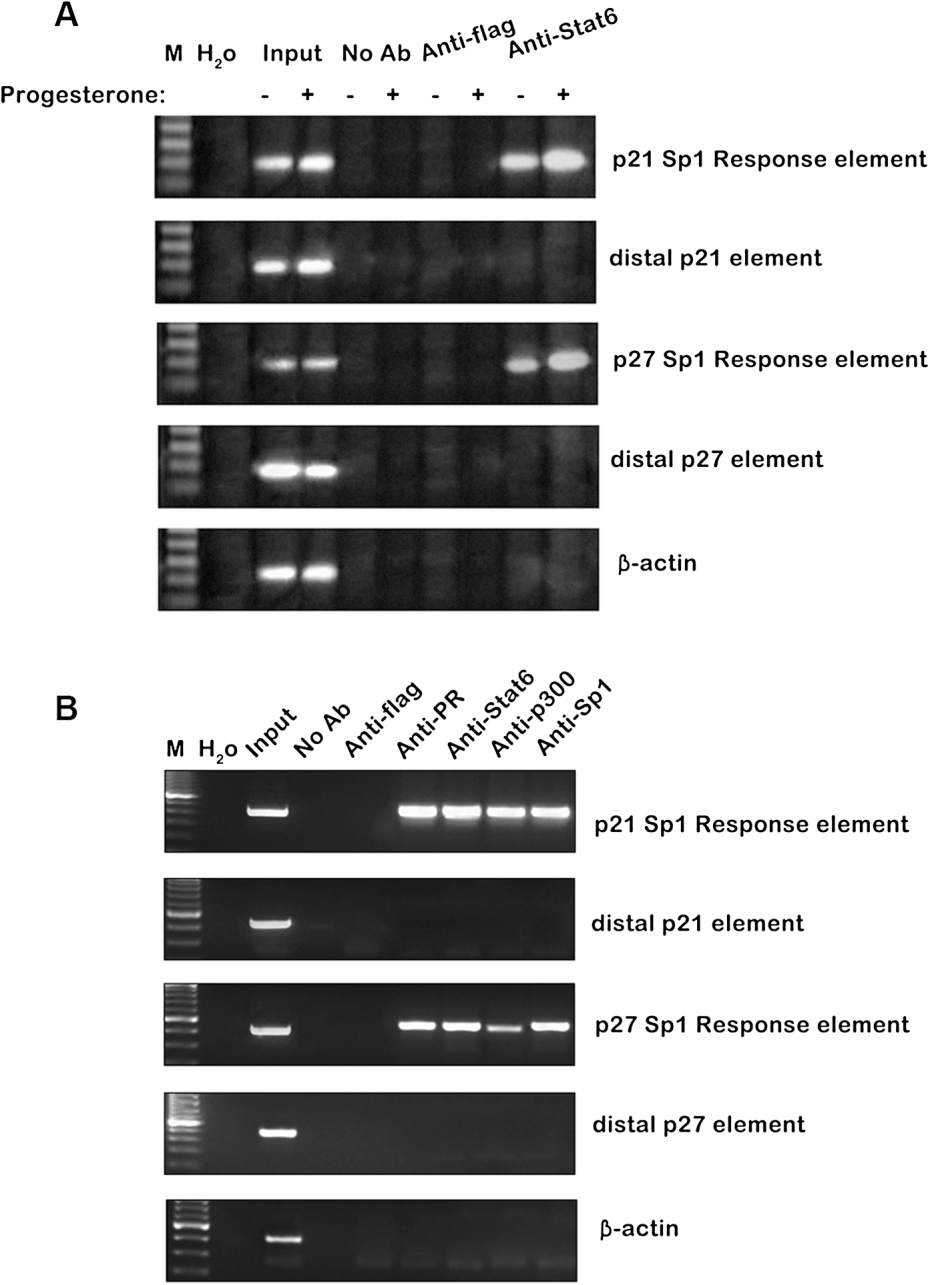
**Progesterone enhances Stat6 recruitment at multiprotein complexes formed with PR and CBP/p300 at proximal Sp1 elements of the p21 and p27 promoters.** Chromatin was prepared from T47D cells incubated with progesterone (30nM) or ethanol (vehicle) **(A)** or with progesterone (30 nM) alone **(B)** for 4 h before lysis. Immunoprecipitations were then performed using antibodies as indicated (top). Controls included PCRs done without DNA (H_2_O) or with nonprecipitated genomic DNA (input) or immunoprecipitation assays performed without antibody (no Ab) or with an irrelevant antibody (anti-flag). The extracted DNA was amplified using the primer pairs covering either the progesterone-responsive Sp1-binding region of the p21 gene promoter (upper panel), a distal region of the p21 gene located ~1 kb from this element, the 544/533 progesterone-responsive Sp1-binding region of the p27 gene promoter, a distal region of the p27 gene located ~1 kb from this element, or a β-actin gene region (lower panel).

To investigate the in-cell occupancy of these Sp1-binding sites by Stat6 and the influence of progesterone on this, chromatin immunoprecipitation assays were performed on DNA isolated from T47D cells either treated with progesterone or untreated (Figure 2A). Consistent with our previous findings in CHO cells (data not shown), Stat6 was found by immunoprecipitation to be associated with the proximal Sp1-binding elements of the p21 and p27 genes. Moreover, this association was greater in the progesterone-treated than in the parallel control cells. Statistical analysis of quantifications of the p21 and p27 promoter sequences bound by Stat6 in ChIP assays are presented in Additional file 2: Figure S2.

Previous reports have indicated that the CBP/p300 protein functions as a coactivator of PR [21,36,37] and cooperates with PR at the proximal Sp1-binding sites of the p21 and p27 gene promoters to increase their activities [19,21]. Consistent with these data, PR, Stat6, and Sp1 were found to be present together with CBP/p300 at the proximal Sp1 elements of the p21 and p27 promoters in progesterone-treated T47D cells (Figure 2B). As a control of specificity, amplification using primers covering regions <1 kb upstream of these sites or oligonucleotides specific for the β-actin gene resulted in non-relevant background products.

In order to determine whether PR, Stat6 and p300 interact with each other and how they are presented in the complex, we conducted systematic immunoprecipitation assays and the results are presented in Additional file 3: Figure S3. Extracts from control or progestrone-treated cells were immunoprecipitated to determine whether Stat6 binds to PR or p300. As demonstrated in Additional file 3: Figure S3, progestrone treatment increased the level of Stat6 in the complexes immunoprecipitated with anti-PR but not in complexes immunoprecipitated with anti-p300. Reprobing the filters with anti-PR and anti-p300 antibodies confirmed that the immunoprecipitates from control and progestrone-treated cells contained the same levels of PR and p300. Therefore, progestrone appears to cause a selective increase in Stat6 binding to PR. The IP and WB assay results confirmed that Stat6 binds to PR. Furthermore, by luciferase assays, Stat6 and PR cooperated to induce P21 and P27 transcriptional activities.

**Figure 3:**
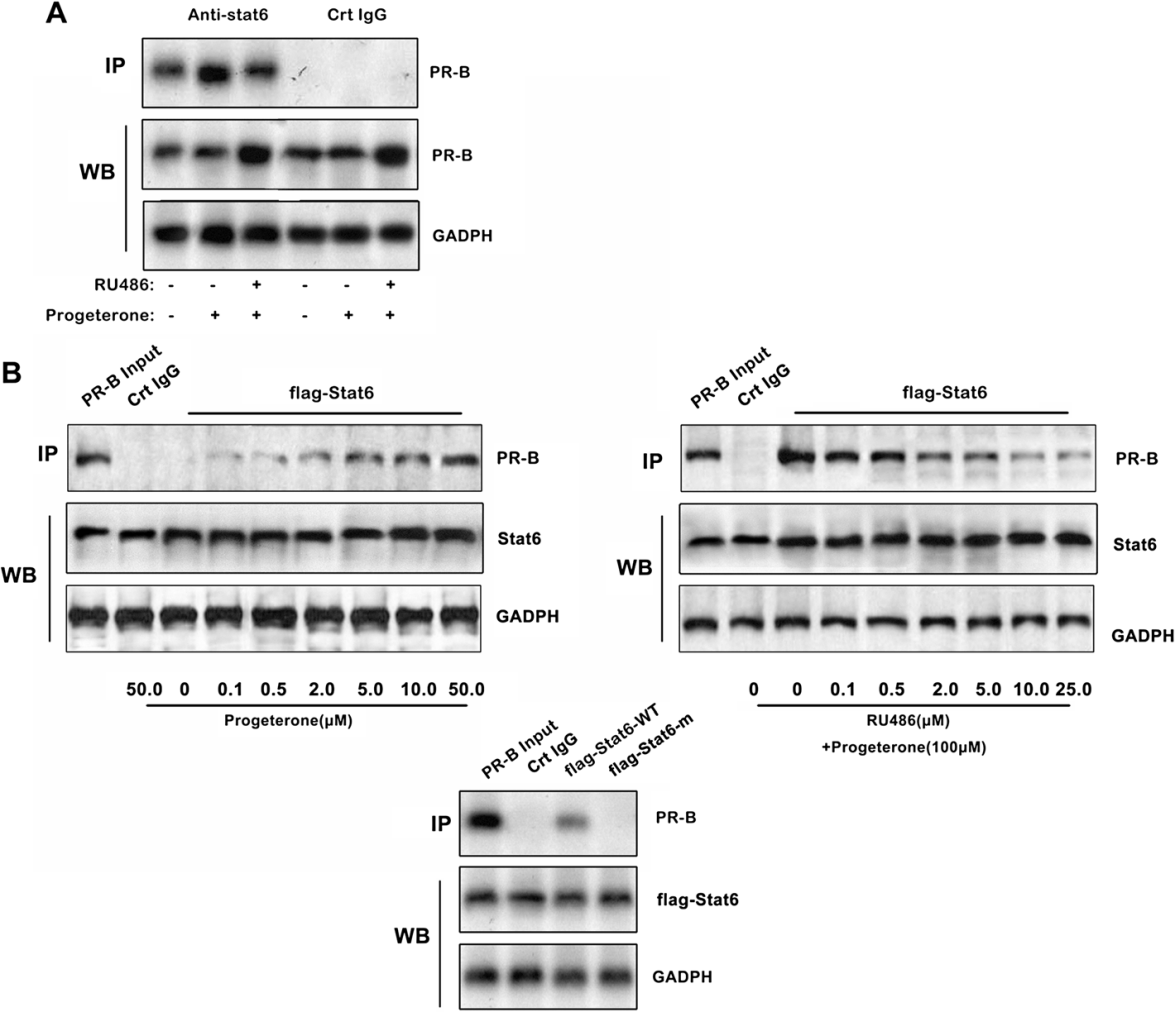
**Stat6 interacts directly with progesterone-bound PR via its LTKLL in the TAD domain. A**. Coimmunoprecipitation assays. T47D cells were treated with progesterone (30 nM) and RU486 (10 nM) for 12 h, or untreated. Total protein extracts (50 μg) were then subjected to Western blotting using a PR antibody either after immunoprecipitation with anti-Stat6 or nonimmune rabbit IgG (negative control) antibodies (upper panel) or directly for control of the in-cell PR levels (lower panel). **B**. as in A, coimmunoprecipitation assays were conducted on cells with the indicated treatment. C, flag–Stat6, either wild type or mutated in the LTKLL (flag-Stat6m) motif, was assayed for interaction with PR as described above in the presence of progesterone (10 nM).

The putative interaction between Stat6 and progesterone-activated PR was then examined. To this end, we first determined in co-immunoprecipitation assays whether endogenous Stat6 and PR interact in T47D cells either untreated or treated for 12 h with progesterone and the partial PR antagonist RU486 (Figure 3A). Low levels of endogenous PR were found in the complex immunoprecipitated with the anti-Stat6 antibody in untreated T47D cells. However, the amount of PR co-immunoprecipitated with Stat6 was drastically increased in cells treated with progesterone alone. This effect was attenuated by co-treatment with RU486 (Additional file 4: Figure S4). From Figure 3A we detected that compared with cells treated with progesterone alone the PR-B protein level was highly enriched by co-treatment with both RU486 and progestrone in western blotting assays, whereas the PR-B protein immunoprecipitated by anti-Stat6 antibody was drastically decreased. RU486 is a well-characterized PR antagonist that binds to the receptor and blocks its gene regulatory function. For RU-486 competitively binds better than progesterone does to the receptor, progesterone is then not able to bind to its own receptor. Although RU486 blocks PR transcriptional activity by favoring corepressors recruitment, it was found that PR turnover was highly reduced after RU486 treatment [10-13]. Like progesterone, RU486 stimulates similar early cascade of events, including chaperone dissociation, dimerization, and posttranslational modifications, such as sumoylation and phosphorylation, which might give explanation of the inconsistency of Stat6 protein levels between western blotting and immunoprecipitaition assays.

To assess further whether Stat6 interacts in vivo with PR, co-immunoprecipitation assays were performed on cells transfected with the flag–Stat6 vector (Figure 3B). The results indicated that Stat6 interacts physically with PR and that this interaction is enhanced in a dose-dependent manner by progesterone (Figure 3B, top left panel). Consistent with Figure 3A, the binding observed between Stat6 and progesterone-activated PR diminished in the presence of RU486 (Figure 3B, top right panel). Indeed, consistent results came from other PR-positive cell lines including MCF-7 (Additional file 5: Figure S5).

Besides, the results of immunoprecipitation assays suggested that Stat6 might act as a downstream target of PR. In order to determine whether there might be PR-responsive elements in the Stat6 promoter or other regulatory elements we have conducted luciferase assays on the cells cotransfected with vectors carrying PRB cDNA and Stat6 promoter sequences and none significant changes have been detect. Consistently, previous study reports no PR binding in the Stat6 promoter in T-47D breast cancer cells[9].

We previously demonstrated that Stat6 carries the transactivation domain (TAD), containing one putative LXXLL motif at amino acids 802 to 806, which are nuclear receptor interaction domains in numerous transcriptional co-regulatory proteins [38]. Interestingly, mutation of the LXXLL motif drastically reduced the binding of Stat6 to PR. This indicates the importance of the LXXLL motif in the *in vivo* interaction between Stat6 and PR (Figure 3B, lower panel). Besides, to further investigate the function of LXXLL motif in Stat6 on the interaction between Stat6 and the p21 or p27 promoter we conducted luciferase assays and RT-PCR assays using a LXXLL-mutant of Stat6, flag-Stat6-m (Additional file 6: Figure S6). Consistently we got that Stat6 suppresses p21 and p27 transcriptional activity, which was abated by flag-Stat6-m transfection.

Taken together, these results suggest that Stat6 is recruited by progesterone-activated PR through its LXXLL motif in TAD in the regulatory complexes formed with Sp1 at the proximal Sp1-binding sites of the p21 and p27 gene promoters.

### Stat6 is required for the progesterone-induced increase of p21 and p27 expression and inhibition of G1/S cell cycle progression

The next question we addressed was whether Stat6 is required to induce p21 and p27 expression as well as in the regulation of cell proliferation by progesterone.

**Figure 4:**
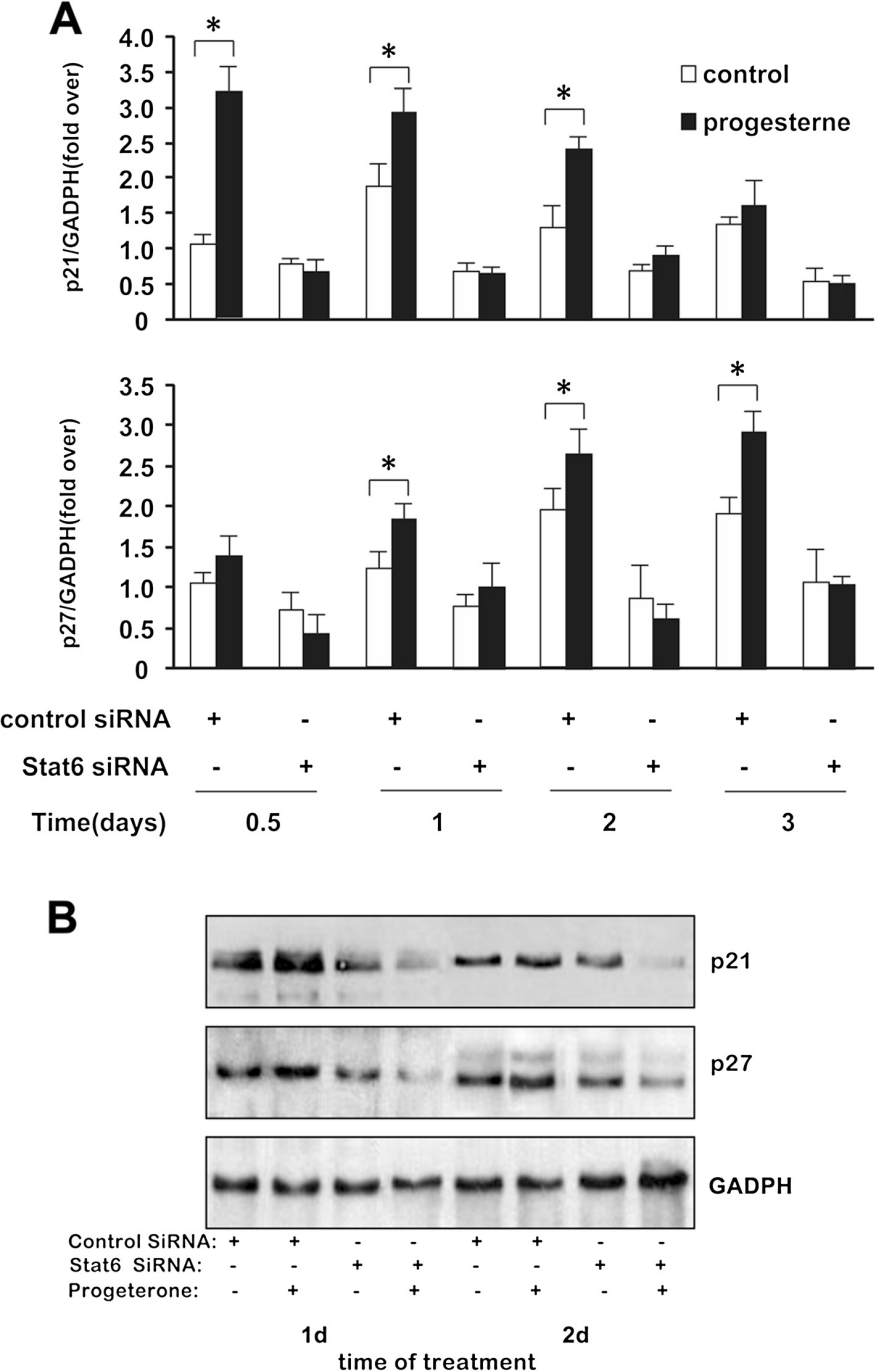
**Stat6 mediates the induction of p21 and p27 gene expression by progesterone.** T47D cells, treated with or without progesterone (30nM) and transfected with control or Stat6 siRNAs (500 ng), were harvested at the indicated times for RNA **(A)** and protein **(B)** extraction. **(A)** Quantitative RT-PCR analyses. mRNA levels are expressed relative to levels in vehicle-treated control siRNA-transfected cells harvested at 24 h, arbitrarily set as 1. *P ≤ 0.05 versus control. **(B)** Western blotting of p21 and p27 with GADPH as control.

First, the expression of both genes in response to progesterone was assessed in T47D cells in which Stat6 expression was silenced using a siRNA strategy (Figure 4). As the results demonstrate, the decrease of Stat6 expression triggered a significant down-regulation of both p21 and p27 mRNA (Figure 4A) and protein (Figure 4B) levels. Moreover, in good agreement with previous observations [19,39], progesterone treatment resulted in an early and transient up-regulation of p21, followed by a delayed and sustained up-regulation of p27. Strikingly, this progesterone-dependent modulation of p21 and p27 gene expression was completely abolished upon siRNA-mediated specific silencing of Stat6.

**Figure 5:**
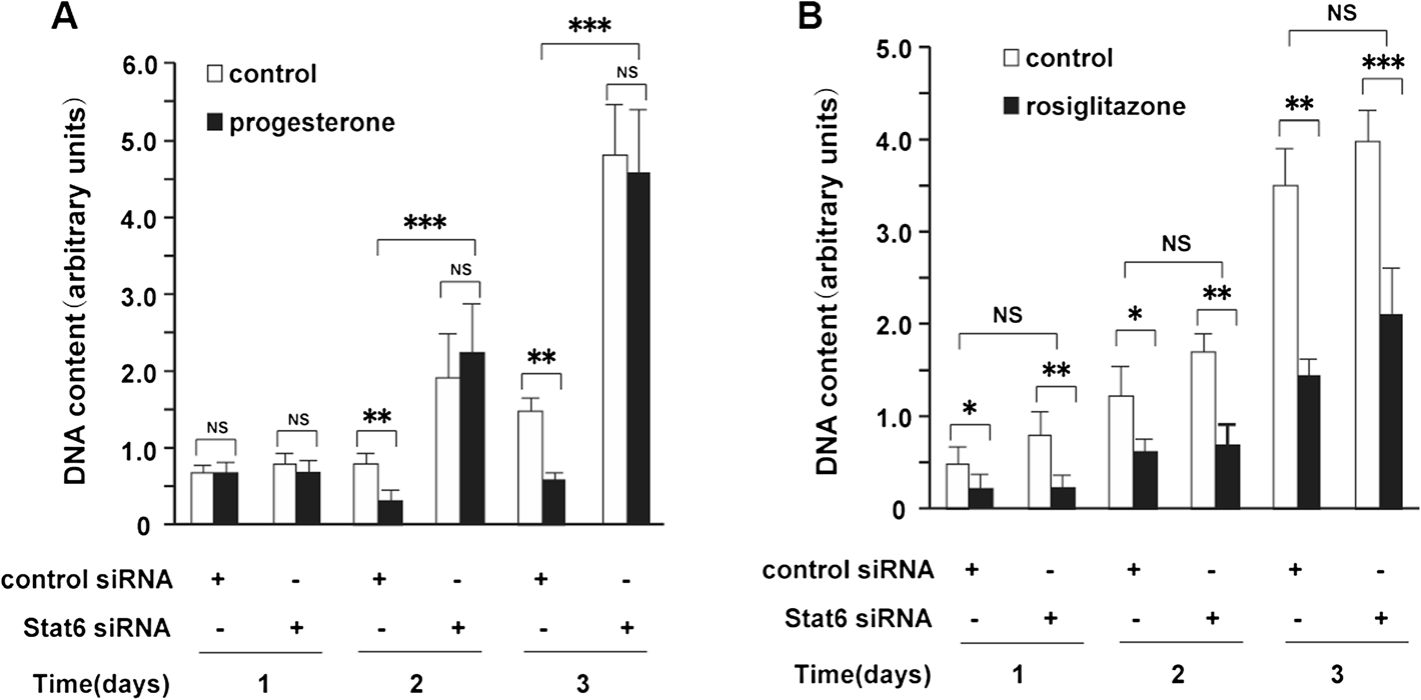
**siRNA silencing of Stat6 abolishes inhibition of T47D cell proliferation by progesterone but not by rosiglitazone.** T47D cells, treated with or without progesterone **(A)** (30 nM) or rosiglitazone **(B)** (0.5 μM) and transfected with control or Stat6 siRNAs (500 ng), were harvested at 1, 2, or 3 days for measurement of DNA content. ns, not significant; *, *P* ≤ 0.05; **, *P* ≤0.01; ***, *P* ≤ 0.001 versus control.

Next, the influence of Stat6 silencing on the growth inhibitory effects of progesterone was tested. Consistent with previous reports, cell growth in the control was inhibited by progesterone after 4 and 6 days of treatment as a consequence of p21 and p27 up-regulation [19,39]. However, siRNA silencing of Stat6 completely prevented the growth-inhibitory effects of progesterone (Figure 5A). To assess the specificity of this effect, the role of Stat6 in the response to rosiglitazone, a ligand of peroxisome proliferator-activated receptor γ (PPARγ), which has previously been reported to inhibit mammary cancer cell growth [40,41], was also examined. In contrast to progesterone, silencing of Stat6 did not alleviate the growth-inhibitory effects of rosiglitazone on T47D cells (Figure 5B). This therefore indicates that Stat6 specifically mediates the inhibition of breast cancer cell proliferation by PR.

**Figure 6:**
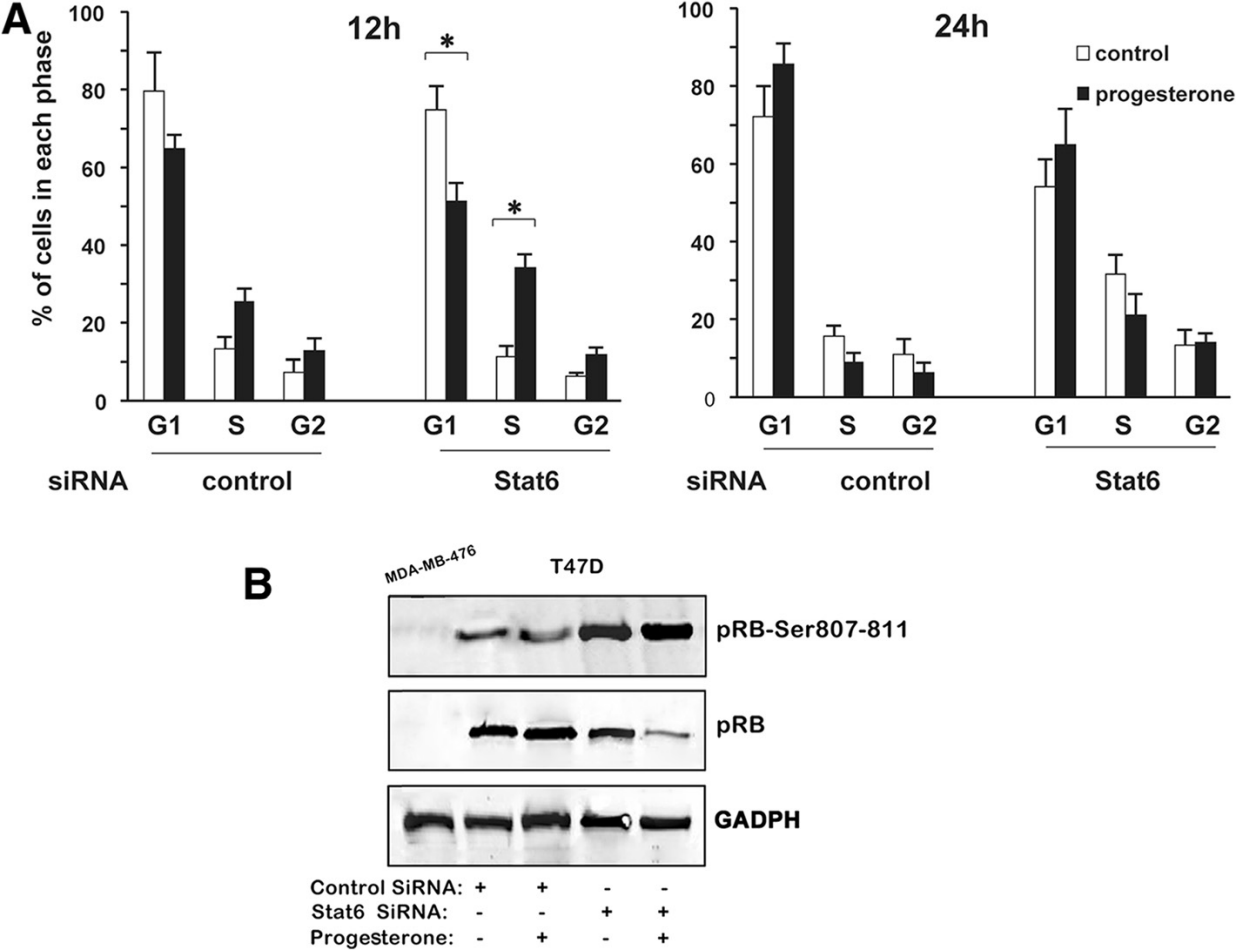
**Stat6 is required for the inhibition of G1/S cell cycle progression by progesterone.** Synchronized T47D cells, transfected with control or Stat6 siRNAs (500 ng) and treated with or without progesterone, were harvested after 12 h or 24 h to determine cell cycle phase distribution **(A)** or Western blotting using either an antibody raised against pRB phosphorylated at Ser807/811 or an antibody recognizing specifically the underphosphorylated form of pRB **(B)**. Representative Western blotting results are shown in B. pRB-Ser807-811, pRB phosphorylated on the Ser807/811 residue; pRB, hypophosphorylated pRB. The MDA-MB-468 cell line, deficient in pRB expression, was used as a negative control of pRB staining.

Finally, the cell cycle phase distribution and in-cell pRB phosphorylation status were analyzed (Figure 6). As previously reported [42,43], progesterone induced an initial acceleration of cell cycle progression (Figure 6A, left panel; performed after 12 h of progesterone treatment), followed by inhibition of the G1/S transition (Figure 6A, right panel; performed after 24 h of progesterone treatment), which was associated with an inhibition of pRB phosphorylation (Figure 6B; performed 24 h after progesterone treatment). Interestingly, siRNA-induced Stat6 silencing did not affect the early cell mitogenic response to progesterone. In contrast, Stat6 knockdown prevented the subsequent decrease in the percentage of cells in S phase and in the regulation of phosphorylated and dephosphorylated pRB levels (Figure 6A, right panel, and B).

Collectively, these data indicate a specific role for Stat6 as a coactivator of PR in the regulation of the progesterone-induced G1-phase cell cycle arrest of breast cancer cells.

### Stat6 mediates the differentiation-enhancing activities of progesterone in breast cancer cells

**Figure 7:**
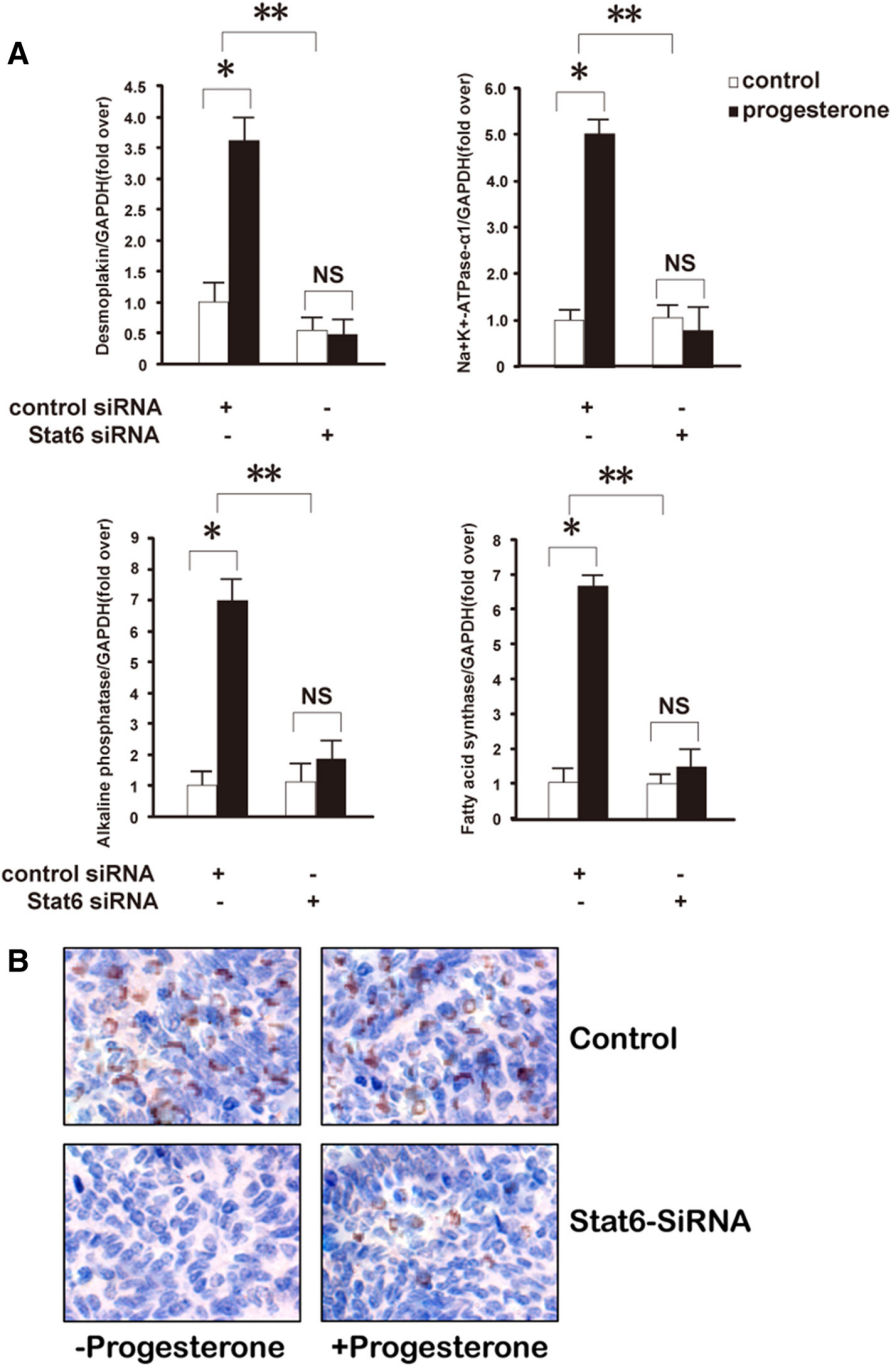
**Stat6 silencing prevents the induction of differentiation by progesterone.** Synchronized T47D cells transfected with control or Stat6 or siRNA (500 ng) and treated with progesterone (30 nM) or vehicle were harvested after 12 h **(A)** or 4 days (**B** and **C**) for quantitative RT-PCR analyses (**A** and **B**) and Oil Red O staining for lipid droplets **(C)**. In panels **A** and **B**, mRNA levels are expressed relative to vehicle-incubated control siRNA-transfected cells, which were arbitrarily set as 1. NS, not significant; *, *P* ≤ 0.05; **, *P* ≤0.01; ***, *P* ≤ 0.001 versus control. **C***.* Lipid detection. Left panel*:* representative Oil Red O-stained cellular sections. Right panel: quantification of the lipid staining intensity.

Recent in vitro studies have associated the ability of progesterone and its derivatives to control mammary cancer cell proliferation negatively by inducing a cell differentiation program [39,44], which leads to the acquisition of a secretory phenotype [45,46]. Therefore, we tested whether siRNA silencing of Stat6 also influences the effects of progesterone on T47D cell differentiation. To this end, the expression levels of a panel of previously identified markers of early and terminal differentiation in breast cancer cells [47,48] were measured (Figure 7A and B). As reported, progesterone induced the early gene expression of desmoplakin and Na^+^/K^+^-ATPase α1, which are markers for epithelial differentiation and glandular development, respectively [47,48] (Figure 7A). Moreover, consistent with previous data [49-51], progesterone increased the expression of FAS (fatty acid synthetase) and ALP (alkaline phosphatase), which are markers of differentiation correlating with lipid storage in breast cancer cells (Figure 7B). Interestingly, the induction of each of these mRNA levels was abolished by Stat6 knockdown (Figure 7A and B). Concurring with these RNA data, progesterone treatment induced an accumulation of lipid droplets as visualized by Oil Red O staining, and this effect was significantly decreased by ~87% in Stat6-deficient cells (Figure 7C).

Therefore, the induction of a differentiated secretory phenotype of breast cancer cells by progesterone requires the expression of Stat6.

### Stat6 gene expression is induced by progesterone

**Figure 8:**
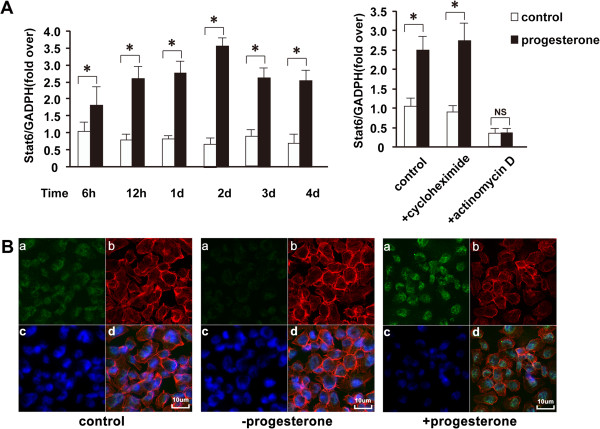
**Stat6 gene expression is induced by progesterone.** Synchronized T47D cells were treated with or without progesterone (30nM) for the indicated times (**A**, left panel) or for 24 h (**A**, right panel, and **B**). **A**. Quantitative PCR analysis. mRNA levels are expressed relative to levels in vehicle-treated control cells harvested at 6 h, arbitrarily set as 1. Right panel. Cycloheximide (5 μg/ml) or actinomycin D (10 μg/ml) was added to the medium 2 h before the addition of progesterone or ethanol (vehicle). h, hour; d, day; NS, not significant; *, *P* ≤ 0.05; **, *P* ≤0.01 versus control. **B**. Modulation of progesterone on Stat6 was detected in T47D cells after 24 h progesterone treatment using Confocal Laser Scanning Microscope. Coverslips seeded with synchronized T47D cells treated with or without progesterone (30nM) and vehicle were triple-stained with Stat6 antibody and one secondary antibody conjugated with FITC to permit detection of Stat6 (a), phalloidin to permit detection of actin (b), and DAPI, a DNA-binding dye, to counterstain the DNA (c). In T47D cells, Stat6 accumulated in nuclear domains that colocalized with dense regions of DAPI staining (d).

Since progesterone exerts biphasic effects on mammary cancer cell proliferation despite the continuous presence of transcriptionally competent PR, it has been proposed that the long-term growth arrest provoked by progesterone requires the induction of additional factors [42,43]. To determine whether Stat6 expression is regulated by progesterone, Stat6 mRNA levels were measured by quantitative RT-PCR analysis in T47D cells following progesterone treatment (Figure 8). Interestingly, treatment with progesterone provoked a long-lasting increase in Stat6 mRNA levels, which was already obvious within 12 h of treatment (Figure 8A, left panel). The induction of Stat6 mRNA expression by progesterone was inhibited by actinomycin D (an inhibitor of RNA polymerase II) but not by cycloheximide (a transcriptional inhibitor of protein synthesis) (Figure 8A, right panel), indicating that progesterone-bound PR could directly induce Stat6 gene transcription. Correlating with the mRNA data, Stat6 protein levels increased in progesterone-treated T47D cells, as shown by Confocal Laser Scanning Microscopy after 24 h of progesterone treatment (Figure 8B). Together, these results indicate that Stat6 is progesterone-responsive gene acting with PR in a positive feedback loop that inhibits mammary cell proliferation and stimulates differentiation.

## Discussion

It has been proposed that the delayed growth arrest provoked by sustained progesterone treatment requires the presence and/or activation of other transcription factors and/or co-regulators acting with PR [46,52]. Both Stat6 and progesterone have previously been shown to act at the G1/S transition checkpoint through similar mechanisms, i.e., transcriptional induction of the p21 and p27 CDKI gene promoters via their proximal Sp1-binding sites [19,21]. Knockdown of p21 and p27 expression using a siRNA approach prevented the growth-inhibitory response to either progesterone or Stat6 ([19,21] (Additional file 7: Figure S7), highlighting their requirements for both inhibitory pathways. Therefore, in the present study, we also investigated possible cross-talk between PR and Stat6 in the control of these cell cycle-regulating genes in the Stat6- and PR-expressing mammary carcinoma T47D cell line. Our results indicate that Stat6 is rapidly recruited following progesterone treatment to the multiprotein complex formed with PR and CBP/p300 at the proximal Sp1-binding elements of the p21 and p27 gene promoters. Moreover, Stat6 and progesterone synergize to transactivate the p21 and p27 gene promoters through these proximal Sp1-binding sites. Co-immunoprecipitation on intact cells and flag –Stat6 vector transfected cells further revealed a physical interaction between PR and Stat6 that was enhanced by progesterone and mediated by the two LXXLL NR-boxes of Stat6. These observations thus identify a novel function for Stat6 as a coactivator of PR implicated in the progesterone-dependent regulation of the p21 and p27 genes.

Basal Stat6 levels could be sufficient to initiate the growth-inhibitory progesterone response via a direct transcriptional effect on the p21 and p27 gene promoters. Indeed, the induction of p21 and p27 observed after 24 h progesterone treatment was inhibited by the RNA polymerase II inhibitor actinomycin D but was not affected by the protein synthesis inhibitor cycloheximide (Additional file 8: Figure S8). However, it was noteworthy that progesterone treatment also provoked an early and steady induction of Stat6 gene expression itself. Although a positive control by progesterone of the expression of PR coactivators such as SRC-1 and CBP/p300 has been observed in normal human endometrium [53], such molecular pathways have not, to our knowledge, yet been demonstrated in breast tissue. Moreover, Stat6 knockdown experiments revealed that Stat6 expression is necessary for the consecutive progesterone-induced transitory p21 and long-lasting p27 increases and the consequent inhibitory effects on G1/S cell cycle progression. These data indicate that Stat6, as both a PR cofactor and a target gene, mediates a positive feedback loop participating in the growth-inhibitory response to progesterone.

Stat6 deficiency did not affect the previously-reported rosiglitazone-induced inhibition of breast cancer cell growth [40,41]. Interestingly, Stat6 siRNA did not affect the transcriptional up-regulation by progesterone of either metallothionein IIA or pepsinogen C, which are genes not directly related to cell cycle control and are regulated by progesterone via a canonical PR-responsive element (5, 63) (Additional file 9: Figure S9). Thus, specific interactions between PR, Stat6, and other proteins included in the multiprotein complexes formed at proximal Sp1-binding elements of the cell cycle regulatory p21 and p27 genes appear to exist and underlie the growth-inhibitory response to progesterone. Importantly, lowering the Sp1 levels by ~70% using a siRNA approach resulted in a significant decrease of p21 and p27 mRNA levels in both the absence and presence of progesterone (Additional file 10: Figure S10). This indicates that Sp1 is required for basal and progesterone-stimulated p21 and p27 promoter activities. However, as shown for the transactivation of the p21 gene by PPARγ [54,55], it is obvious that a contribution of Sp3 and/or Sp4 protein to the regulation of the p21 and/or p27 promoters by Stat6 and progesterone cannot be excluded. Besides, in addition to the LXXLL motif of TAD, the primary structure of Stat6 contains several other putative interaction domains with co-regulatory proteins [56,57]. Significantly, Stat6 participates in transcriptional regulation by interacting with CBP/p300 [58,59], which is, to date, the only known PR coactivator that synergizes with progesterone-bound PR to transactivate the p21 and p27 promoters [19,21]. Whether additional transcriptional regulatory factors such as CBP/p300 also interact with Stat6 to mediate the progesterone response therefore remains to be investigated.

However, it is possible that Stat6 binds to nuclear receptors or cofactors at Sp1 sites of cell cycle regulatory genes to regulate their expression in contexts other than the progesterone response. In fact, the proximal Sp1-binding regions of the p21 and p27 gene promoters integrate different growth modulatory signals. For instance, the Sp1 sites are involved in the transactivation of the p21 promoter by transforming growth factor [60] and BRCA1, which is also a co-repressor for the estrogen receptor, the androgen receptor, and the PR [61,62]. The proximal Sp1 sites of the p27 promoter are required for responses to vitamin D3 [63] and tamoxifen [64]. Furthermore, the Sp1-binding sites have a common important role in modulating the cell cycle [65,66]. Notably, they also mediate the induction of cyclin D1 by estrogens [67]. Thus, further studies are warranted in order to determine whether Stat6 could play a more general role in the cross-talk among different growth signaling pathways.

Our present data demonstrate that Stat6 itself acts as a differentiation factor in breast epithelial cancer cells, notably in the mediation of progesterone effects. Indeed, the presence of Stat6 was a prerequisite for the induction of cell differentiation by progesterone, as well as for the progesterone-dependent increase in the synthesis of lipid droplets, which is associated with a differentiated secretory phenotype in mammary cells [49,50]. It is tempting to propose that p21 and p27 are involved in the Stat6-dependent effects on breast cancer cell differentiation. In fact, the induction of p21 and p27 has been shown to constitute a molecular switch that facilitates hormone-induced differentiation in numerous cell systems [20,68]. In breast tissue, high levels of p21 and p27 are found in intermediately differentiated and well-differentiated ductal carcinoma in situ, respectively [69]. Furthermore, p27 up-regulation is thought to be the molecular basis for the blockage in the alveolar differentiated state [70]. Thus, the molecular mechanisms underlying the function of Stat6 in mammary cell differentiation and the putative role of p21 and p27 induction therein remain to be investigated.

The role of PR activity on breast tumorigenesis has been a subject of controversy. In epidemiological studies, progesterone levels during a first pregnancy at an early age are thought to confer protective effects against future breast tumor development [71,72]. However, the possible deleterious effect of certain progestins used in hormone replacement therapies (HRTs) after menopause and as contraceptives to counteract the proliferative action of unopposed estrogens on the uterus recently resurfaced in the Women’s Health Initiative and the Million Women studies [73,74]. These studies showed that a combined estrogen plus progestin regimen as HRT is associated with a greater risk of breast cancer than estrogen alone or placebo. In spite of these data, different reports have also indicated that the effects of progesterone and its derivatives could depend on several different factors, including the family history of breast cancer, the mode of administration, and the dose and type of progestin [75,76]. The delayed action of progesterone derivatives on breast cancer cell growth could explain their potential differential stimulatory or growth-inhibitory effects, depending on whether the treatment is transitory or continuous, respectively [11-14]. Interestingly, siRNA knockdown of Stat6 did not modulate the influence of progesterone on the initial proliferative burst, as shown by FACS analysis (Figure 6A, left panel) or proliferation assays (Additional file 11: Figure S11). In agreement with this, the induction of expression of the cyclin D1, D3, A, B1, or E genes, which participate in cell cycle progression [77,78], was not affected by Stat6 siRNA transfection after 12 h of progesterone treatment (Additional file 12: Figure S12). These data, indicating a role for Stat6 specifically in the growth-inhibitory response to progesterone, suggest that the effects of each progestin treatment could also depend on levels of Stat6 expression in breast epithelium and/or of Stat6 recruitment to the PR.

In clinical practice, the expression of PR in breast tumor biopsies is assessed as a predictive marker for favorable disease prognosis, with the absence of PR reflecting a nonfunctional estrogen receptor and resistance to hormone therapy [79,80]. Expression levels of PR correlate with p27 and differentiation status in large populations of primary mammary tumors [18,81]. p27 mRNA and protein levels in breast cancer biopsies correlate positively with a favorable outcome in human breast tumors, whereas a loss of p27 gene expression is associated with a shorter overall survival [82-84]. However, there is a subgroup of steroid receptor-positive tumors with low levels of p27, which indicate poor prognosis and are resistant to antiestrogen therapy [85]. It is tempting to speculate that altered activity of transcriptional factors, such as Stat6, involved with PR in the (dys)regulation of the p27 gene promoter, could play a role in p27 loss and mammary tumor development. It is noteworthy that Stat6 activities affect apoptosis and gene expression in breast cancer cells [86]. Moreover, the human Stat6 gene lies on the chromosome 12q13.3-q14.1 locus [87]. The recently published Cancer Genome Atlas (TCGA) data set [88] includes high-resolution information on DNA copy number, mRNA and microRNA expression, DNA methylation, single-nucleotide polymorphisms, and somatic mutations in cancer. One of the most common copy-number alterations in breast cancer is amplification at chromosome 12q13.3–14.1, which is also amplified in melanoma and lung cancers [89]. The *CDK4* gene has been proposed as the target of this amplification. CDK4 promotes proliferation by inhibiting the RB1 tumor suppressor and by sequestering p27Kip1 and p21Cip1, thereby promoting E2F- and Cdk2-dependent cell cycle progression [90]. However, CDK4 overexpression alone does not induce spontaneous tumorigenesis in transgenic animal models, suggesting that CDK4 cooperates with other genetic changes during tumorigenesis [90]. Analyses of potential correlations between genetic alterations of Stat6, CDK4 and p27 expression could be decisive in clarifying the role of Stat6 in familial predisposition to certain cancers and/or failure of hormonal therapy.

Following tamoxifen, the first identified selective estrogen receptor modulator, a number of other antiestrogens have been developed [91]. The notion that selective nuclear receptor modulators can exhibit cell- and tissue-specific effects has also been extended to a panel of other nuclear receptors [92]. Selective progesterone receptor modulators, such as Asoprinil, are now under investigation in the treatment of uterine fibroids and endometriosis [93]. However, to date, they have mostly been identified by empirical and in vitro studies [94]. The characterization of Stat6 as a PR coregulator molecule involved in the antiproliferative and differentiation effects of progesterone in breast tissue could potentially explain the biological effects of different progestins on breast cancer and guide the future discovery of drugs. In fact, selective progesterone receptor modulators inducing Stat6 cofactor recruitment to PR could prevent breast cancer development when used in HRT, contraceptives, or treatment of uterine diseases. Further studies are thus warranted to evaluate the putative tissue-specific roles of Stat6 in progesterone pathways.

## Conclusions

*In vitro* studies have demonstrated a biphasic progesterone response, consisting of an initial proliferative burst followed by a sustained growth arrest. Our present study demonstrates that Stat6 induces p21 and p27 gene expression by synergizing with progesterone-bound PR at the proximal Sp1-binding sites in the p21 and p27 gene promoters. Stat6 knockdown abolished the inhibitory effects of progesterone on pRB phosphorylation, G1/S cell cycle progression, cell proliferation and breast cell differentiation. Taken together, these data identify Stat6 as a coactivator of PR mediating the growth-inhibitory and differentiation effects of progesterone on breast cancer cells.

## Abbreviations

PR: Progesterone receptor; NR: Nuclear receptor; pRB: Retinoblastoma gene product; CDK: Cyclin-dependent kinase; CDKI: Cyclin-dependent kinase inhibitor; Stat6: Signal transducer and activator of transcription 6; FAS: Fatty acid synthase; ALP: Alkaline phosphatase; PPARγ: Peroxisome proliferator-activated receptor γ.

## Competing interests

The authors declare that they have no competing interests.

## Authors’ contributions

MW and QH designed the study, applied for funding and drafted the manuscript. BYL participated in study design and manuscript preparation, and managed the study. YZY, QZ and ZWW carried out experiments and ensured protocol integrity and collected data. QLG and LPS designed the study, undertook statistical analysis and assisted with drafting of the manuscript. YYY, GFZ and ZGZ conceived the study, participated in its design and coordination, and helped to draft the manuscript. All authors read and approved the final manuscript.

## Pre-publication history

The pre-publication history for this paper can be accessed here:

http://www.biomedcentral.com/1471-2407/14/10/prepub

## Supplementary Material

Additional file 1: Figure S1 Western blotting analysis on Stat6-siRNAs treated T47D cells. Representative experiments have been performed with Stat6 siRNA-3. Mock and nonsence control (ns) were used as negative controls. Click here for file

Additional file 2: Figure S2 Statistic analysis on the quantifications of the p21 and p27 promoter sequences bound by Stat6 in Figure 2A. *, P < 0.05. Columns, mean of three experiments; bars, SD. Click here for file

Additional file 3: Figure S3 Progestrone treatment increased the level of Stat6 in the complexes immunoprecipitated with anti- PR but not with anti-p300. T47D cells were treated with (+) or without (-) 50 ng progestrone for 24 h. Protein extracts were immunoprecipitated with anti-PR or anti-P300 antibodies. Normal rabbit IgG was used as the control. The PR-or P300-associated Stat6 in the resultant immune complexes was analyzed by western blotting using anti-Stat6 antibody with anti-PR or -P300 antibody as loading controls. Click here for file

Additional file 4: Figure S4 Statistic analysis on the quantifications of the band intensity of PR-B immunoprecipitated by Stat6. *, P < 0.05. Columns, mean of three experiments; bars, SD. Click here for file

Additional file 5: Figure S5 Stat6 interacts directly with progesterone-bound PR via its LTKLL in the TAD domain. A. Coimmunoprecipitation assays. MCF-7 cells were treated with progesterone (30 nM) and RU486 (10 nM) for 12 h, or untreated. Total protein extracts (50 μg) were then subjected to Western blotting using a PR antibody either after immunoprecipitation with anti-Stat6 or nonimmune rabbit IgG (negative control) antibodies (upper panel) or directly for control of the in-cell PR levels (lower panel). B, flag–Stat6, either wild type or mutated in the LTKLL (flag-Stat6m) motif, was assayed for interaction with PR as described above in the presence of progesterone (10 nM). Click here for file

Additional file 6: Figure S6 The LXXLL motif of Stat6 is required for Stat6 to transcriptionally modulate p21 and p27. A, The LXXLL motif in Stat6 is mutated as indicated. B, Transcriptional activity analysis of wild-type or mutated Stat6 fusion protein in T47D cells. Each construct containing wild type or mutated Stat6 fusion protein was transiently transfected along with P21Luc or P27Luc plasmid into cultured cells and assayed for luciferase activity. Luciferase activity was normalized to activities of the empty vector of pGL3-luc, expressed as fold difference. Transfections were done in three individual experiments. Bars, SD. P ≤0.05 was considered significant. C, results of q-RT-PCR analyses of Stat6, flag-Stat6-m and p21 and p27 confirm the induction of p21 and p27 by flag-Stat6-WT. Transcriptional activities of p21 and p27 were inhibited by flag-Stat6-m transfection in T47D cells. Expression levels of selected genes (X axis) analyzed by q-RT-PCR were quantified. The Y axis represents the gene expression level normalized to GAPDH for cells transiently transfected with the indicated plasmids for 24 h. These results represent at least three RNA samples per experimental condition run in triplicate. Click here for file

Additional file 7: Figure S7 Overexpression Stat6 abolishes promotion of T47D cell proliferation by p21 and p27 siRNA. T47D cells treated with p21 (700 ng) (left panel), p27 (500 ng) (right panel) and Stat6-flag vector (500 ng) and their parallel controls, were harvested at 1, 2, or 3 days for measure of DNA content. ns, not significant; *, P < 0.05; **, P < 0.01 versus control. Click here for file

Additional file 8: Figure S8 The induction of p21 and p27 observed after 24 h progesterone treatment was inhibited by actinomycin D but was not affected by cycloheximide. Synchronized T47D cells were treated with or without progesterone (30 nM) for 24 h. mRNA levels are analyzed using quantitative PCR analysis. Cycloheximide (5ug/ml) or actinomycin D (10ug/ml) was added to the medium 1 h 30 min before the addition of progesterone or ethanol (vehicle). NS, not significant; *, P < 0.05 versus control. n = 3. (TIFF 1112 kb) Click here for file

Additional file 9: Figure S9 Stat6 siRNA did not affect either the transcriptional up-regulation by progesterone of metallothionein IIA (left panel) and pepsinogen C (right panel). T47D cells transfected with control or Stat6 siRNAs (500 ng), were harvested at the indicated times for RNA and undergone Quantitative RT-PCR analyses on metallothionein IIA and pepsinogen C mRNA. mRNA levels are expressed relative to levels in control siRNA-transfected cells harvested at 24 h, arbitrarily set as 1. *P ≤ 0.05 versus control. Click here for file

Additional file 10: Figure S10 Lowering of Sp1 levels resulted in a significant decrease of p21 and p27 mRNA levels in both the absence (A) and presence of progesterone (B).T47D cells transfected with control or Sp-1 siRNA (500 ng), were harvested at the indicated times for RNA and undergone Quantitative RT-PCR analyses on p21 and p27 mRNA. mRNA levels are expressed relative to levels in vehicle-treated control siRNA-transfected cells harvested at 24 h, arbitrarily set as 1. *P ≤ 0.05; **, P < 0.01 versus control. Click here for file

Additional file 11: Figure S11 Knocking-down of Stat6 did not modulate the influence of progesterone on the initial proliferative burst as shown by proliferation assays. T47D cells transfected with control or Stat6 siRNA (500 ng) for indicated time, were harvested and undergone proliferation analyses.*P ≤ 0.05 versus control. Click here for file

Additional file 12: Figure S12 Cell cycle modulators which participate in G1/S cell cycle progression were not affected by Stat6 siRNA transfection after progesterone treatment. After 12 h of progesterone pretreatment T47D cell were transfected with Scrambled siRNA/Stat6 siRNA for 24 h followed by western blotting analysis. Protein profile analysis was conducted over a time course. Click here for file
