# Supplementary figures and images for: Integrity of the LXXLL motif in Stat6 is required for the inhibition of breast cancer cell growth and enhancement of differentiation in the context of progesterone
Source: BMC Cancer. 2014 Jan 8;14:10. doi: 10.1186/1471-2407-14-10 (PMC4021501; doi:10.1186/1471-2407-14-10)

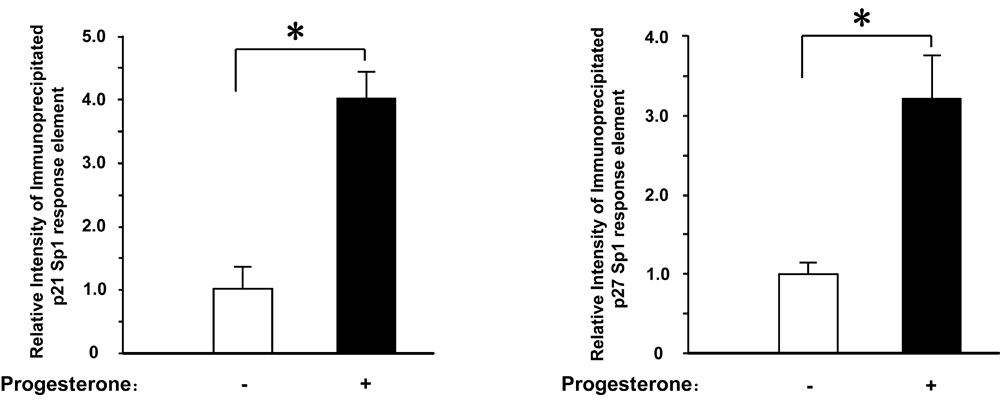

Supplement: Additional file 1: Figure S1 — Western blotting analysis on Stat6-siRNAs treated T47D cells. Representative experiments have been performed with Stat6 siRNA-3. Mock and nonsence control (ns) were used as negative controls. [file 1471-2407-14-10-S1.tiff]

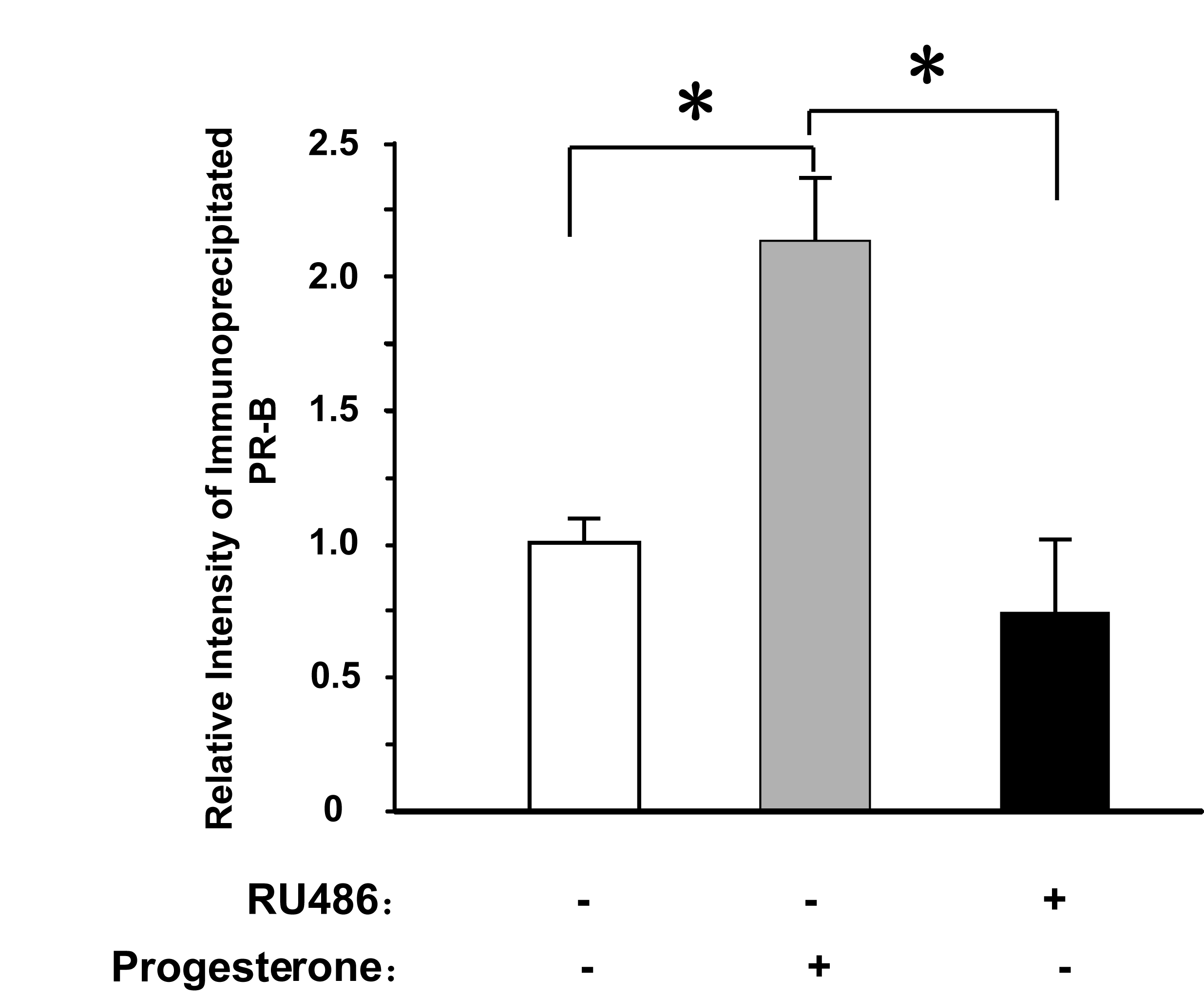

Supplement: Additional file 2: Figure S2 — Statistic analysis on the quantifications of the p21 and p27 promoter sequences bound by Stat6 in Figure 2A. *, P < 0.05. Columns, mean of three experiments; bars, SD. [file 1471-2407-14-10-S2.tiff]

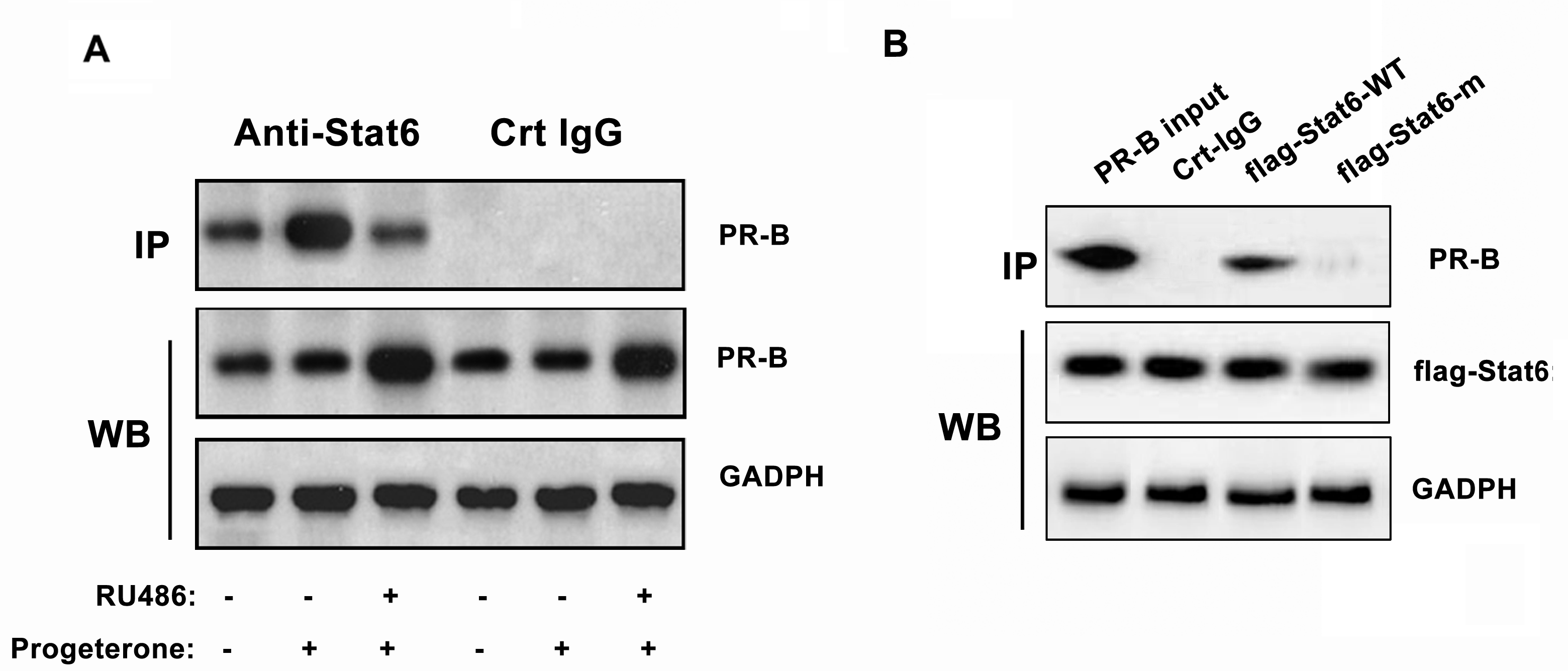

Supplement: Additional file 3: Figure S3 — Progestrone treatment increased the level of Stat6 in the complexes immunoprecipitated with anti- PR but not with anti-p300. T47D cells were treated with (+) or without (-) 50 ng progestrone for 24 h. Protein extracts were immunoprecipitated with anti-PR or anti-P300 antibodies. Normal rabbit IgG was used as the control. The PR-or P300-associated Stat6 in the resultant immune complexes was analyzed by western blotting using anti-Stat6 antibody with anti-PR or -P300 antibody as loading controls. [file 1471-2407-14-10-S3.tiff]

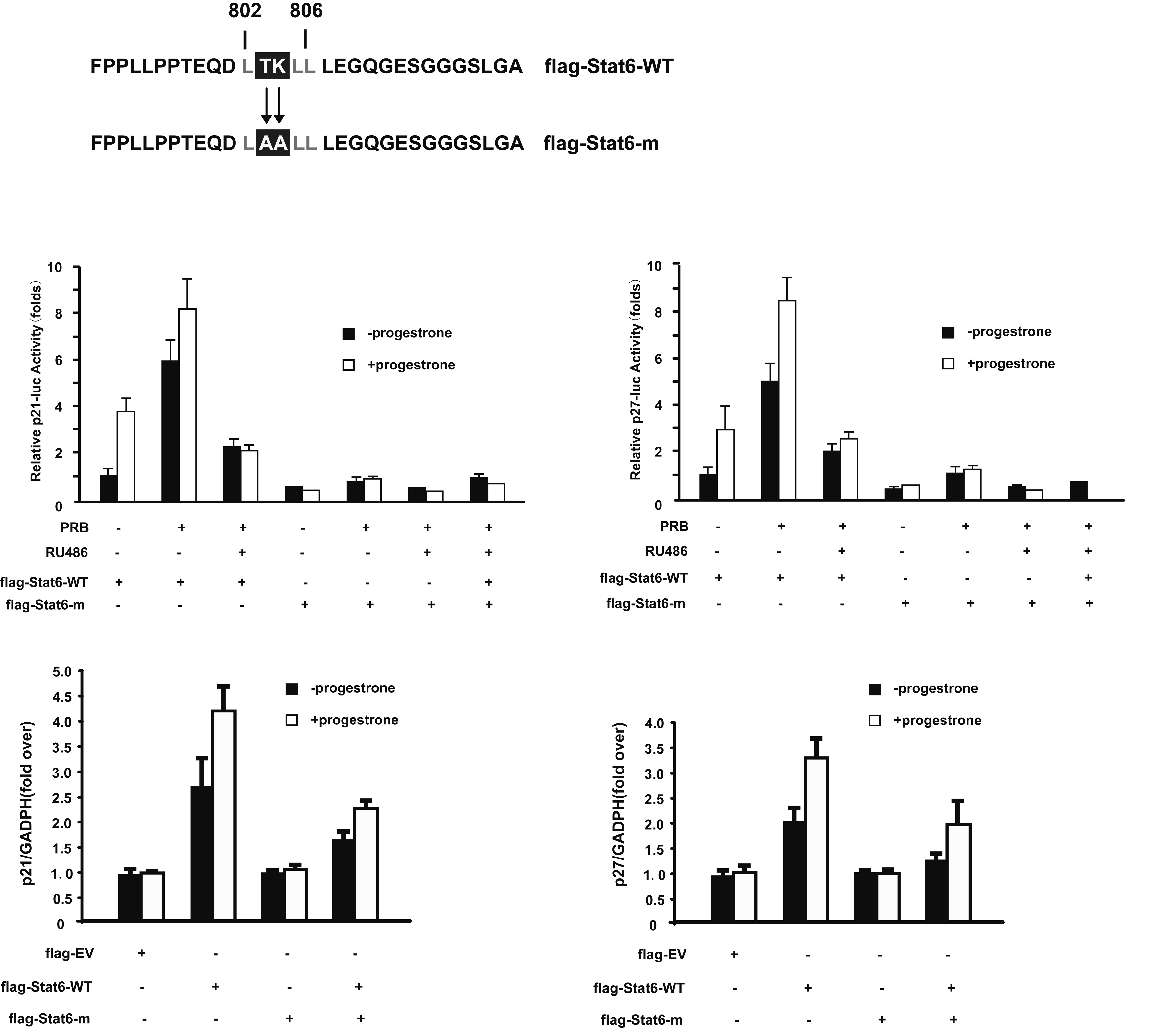

Supplement: Additional file 4: Figure S4 — Statistic analysis on the quantifications of the band intensity of PR-B immunoprecipitated by Stat6. *, P < 0.05. Columns, mean of three experiments; bars, SD. [file 1471-2407-14-10-S4.tiff]

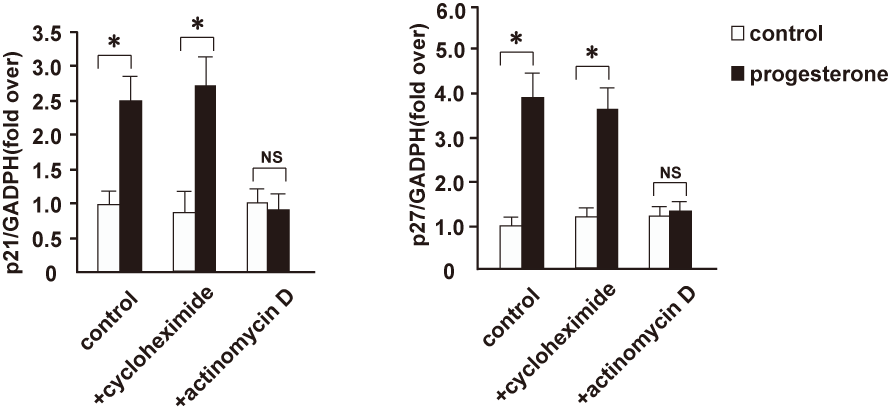

Supplement: Additional file 5: Figure S5 — Stat6 interacts directly with progesterone-bound PR via its LTKLL in the TAD domain. A. Coimmunoprecipitation assays. MCF-7 cells were treated with progesterone (30 nM) and RU486 (10 nM) for 12 h, or untreated. Total protein extracts (50 μg) were then subjected to Western blotting using a PR antibody either after immunoprecipitation with anti-Stat6 or nonimmune rabbit IgG (negative control) antibodies (upper panel) or directly for control of the in-cell PR levels (lower panel). B, flag–Stat6, either wild type or mutated in the LTKLL (flag-Stat6m) motif, was assayed for interaction with PR as described above in the presence of progesterone (10 nM). [file 1471-2407-14-10-S5.tiff]

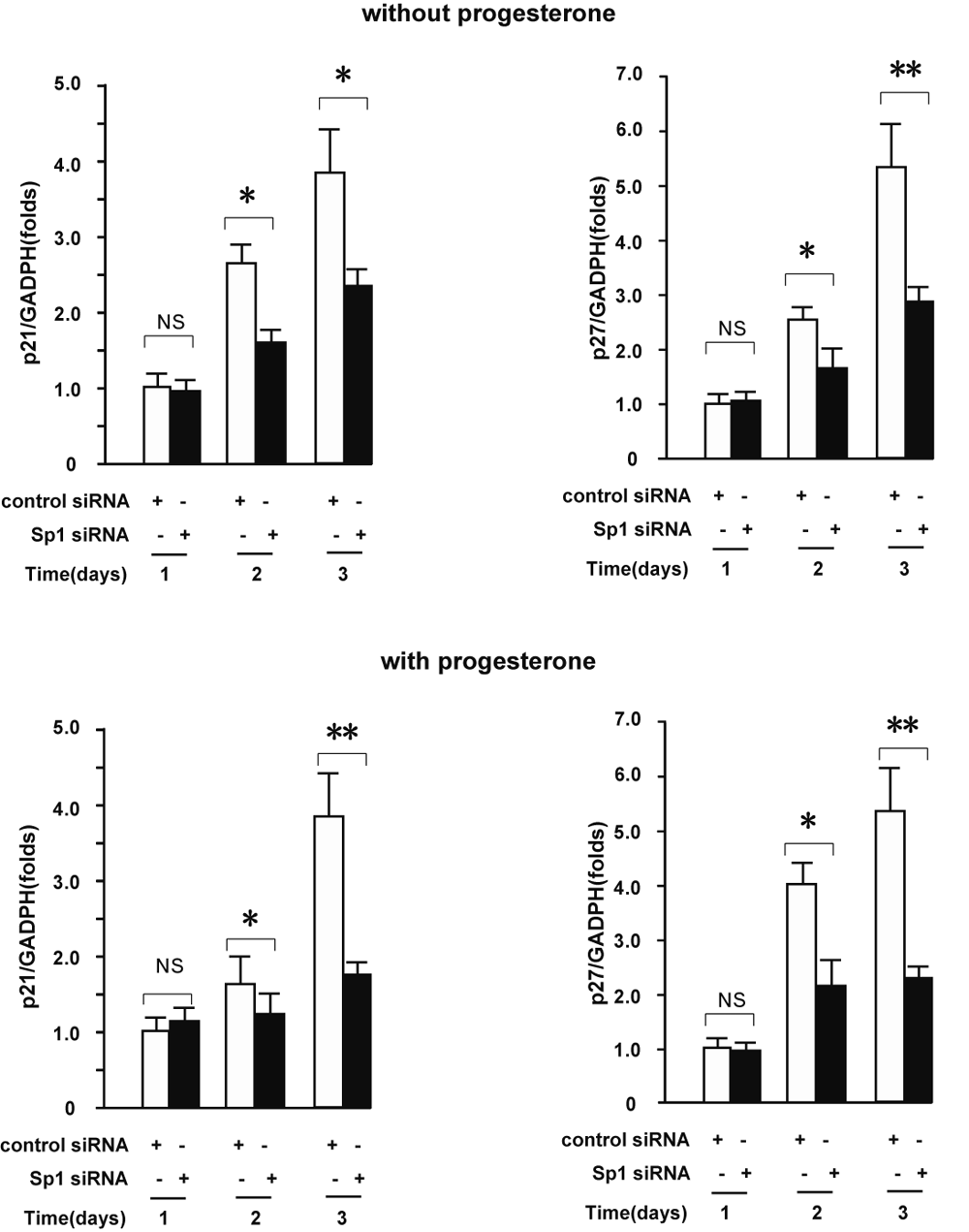

Supplement: Additional file 6: Figure S6 — The LXXLL motif of Stat6 is required for Stat6 to transcriptionally modulate p21 and p27. A, The LXXLL motif in Stat6 is mutated as indicated. B, Transcriptional activity analysis of wild-type or mutated Stat6 fusion protein in T47D cells. Each construct containing wild type or mutated Stat6 fusion protein was transiently transfected along with P21Luc or P27Luc plasmid into cultured cells and assayed for luciferase activity. Luciferase activity was normalized to activities of the empty vector of pGL3-luc, expressed as fold difference. Transfections were done in three individual experiments. Bars, SD. P ≤0.05 was considered significant. C, results of q-RT-PCR analyses of Stat6, flag-Stat6-m and p21 and p27 confirm the induction of p21 and p27 by flag-Stat6-WT. Transcriptional activities of p21 and p27 were inhibited by flag-Stat6-m transfection in T47D cells. Expression levels of selected genes (X axis) analyzed by q-RT-PCR were quantified. The Y axis represents the gene expression level normalized to GAPDH for cells transiently transfected with the indicated plasmids for 24 h. These results represent at least three RNA samples per experimental condition run in triplicate. [file 1471-2407-14-10-S6.tiff]

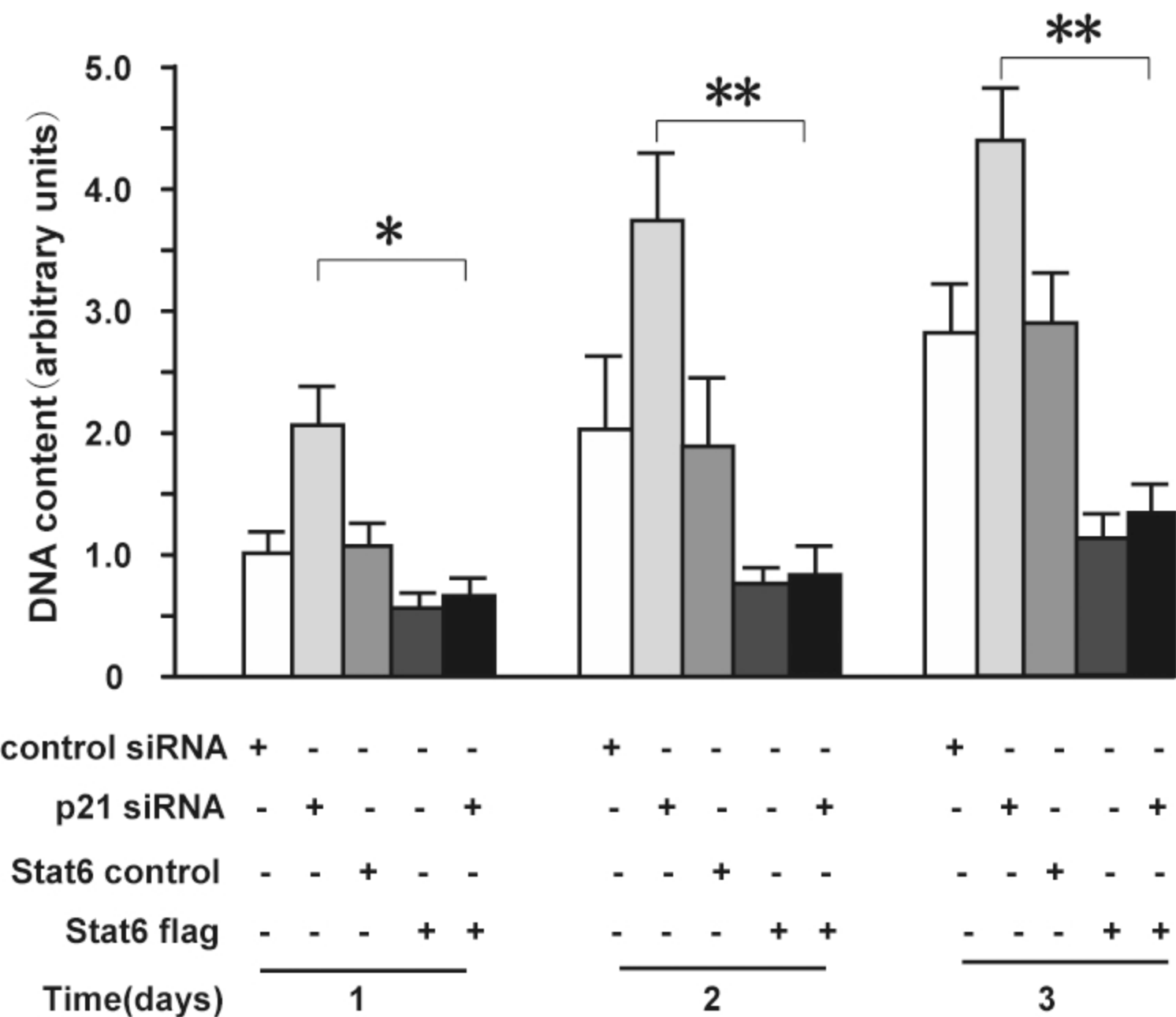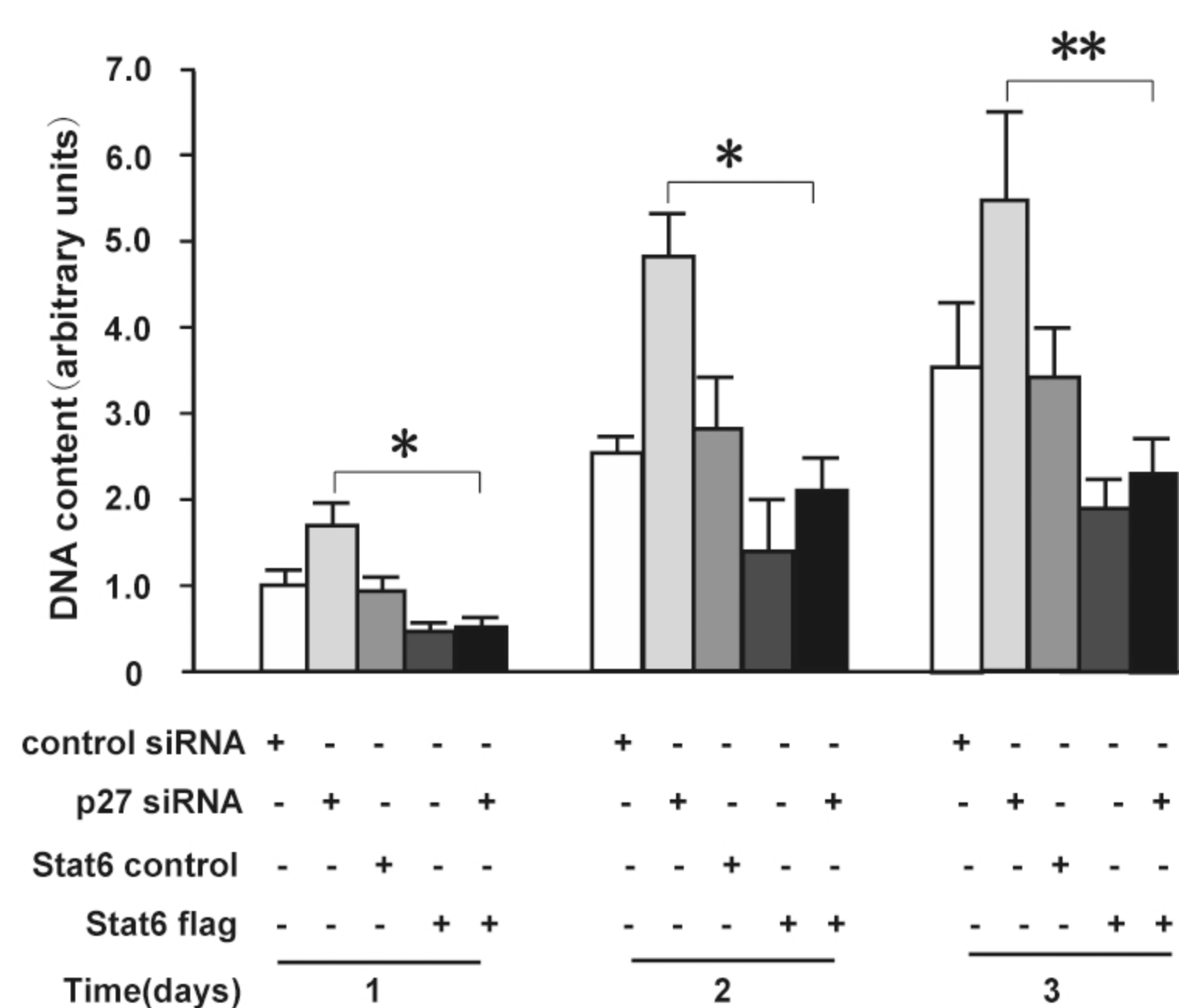

Supplement: Additional file 7: Figure S7 — Overexpression Stat6 abolishes promotion of T47D cell proliferation by p21 and p27 siRNA. T47D cells treated with p21 (700 ng) (left panel), p27 (500 ng) (right panel) and Stat6-flag vector (500 ng) and their parallel controls, were harvested at 1, 2, or 3 days for measure of DNA content. ns, not significant; *, P < 0.05; **, P < 0.01 versus control. [file 1471-2407-14-10-S7.pdf]

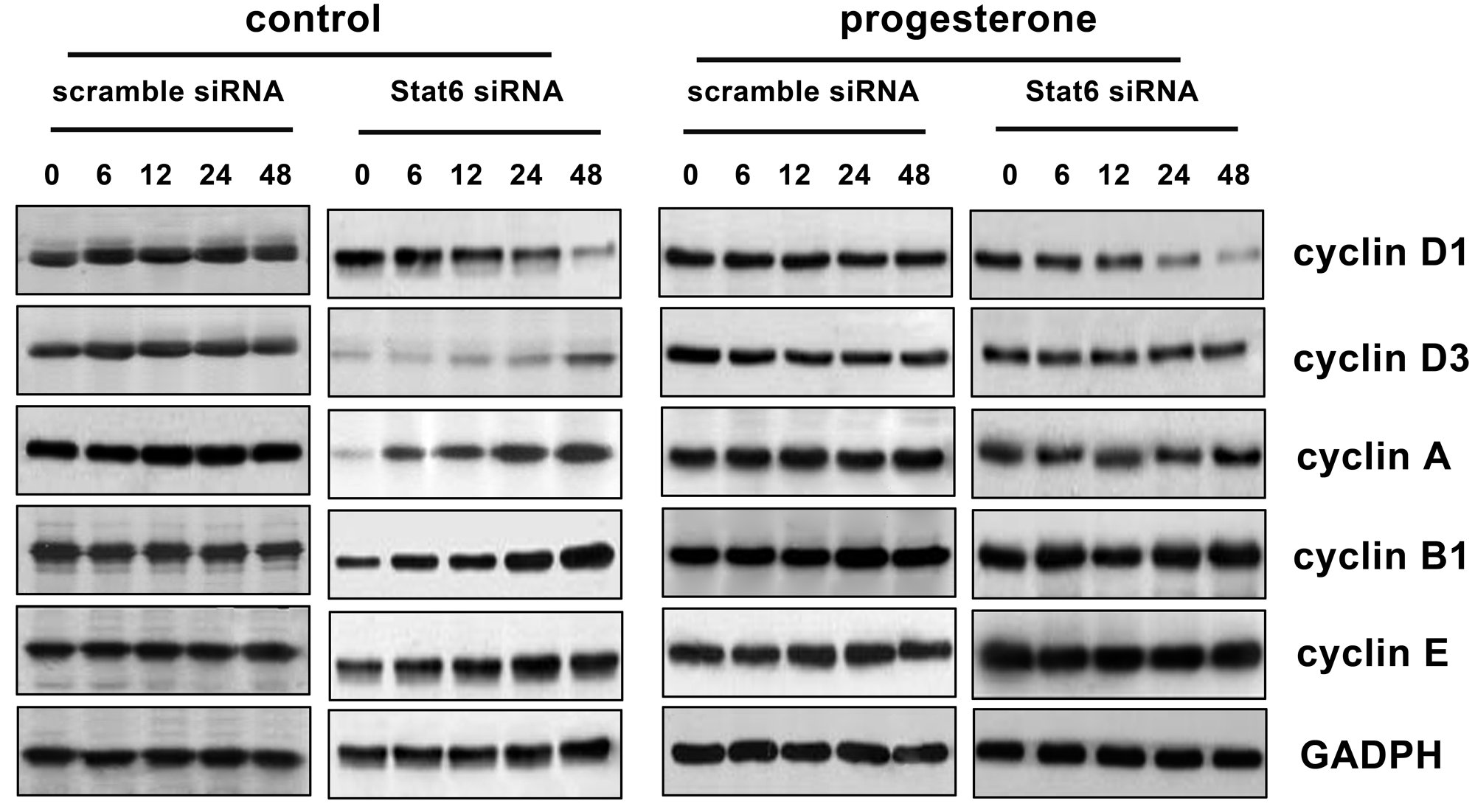

Supplement: Additional file 8: Figure S8 — The induction of p21 and p27 observed after 24 h progesterone treatment was inhibited by actinomycin D but was not affected by cycloheximide. Synchronized T47D cells were treated with or without progesterone (30 nM) for 24 h. mRNA levels are analyzed using quantitative PCR analysis. Cycloheximide (5ug/ml) or actinomycin D (10ug/ml) was added to the medium 1 h 30 min before the addition of progesterone or ethanol (vehicle). NS, not significant; *, P < 0.05 versus control. n = 3. (TIFF 1112 kb) [file 1471-2407-14-10-S8.tiff]

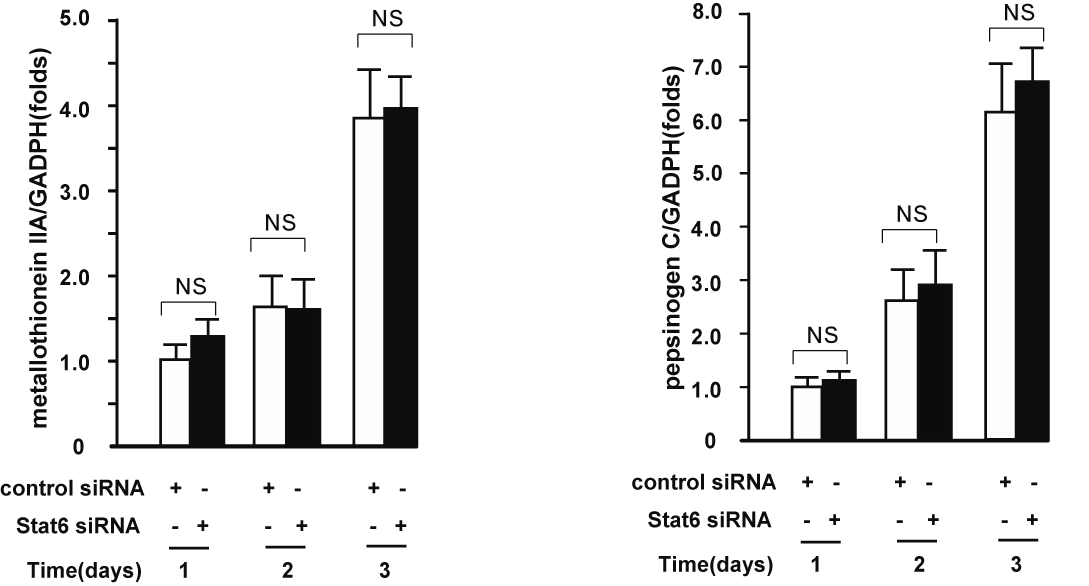

Supplement: Additional file 9: Figure S9 — Stat6 siRNA did not affect either the transcriptional up-regulation by progesterone of metallothionein IIA (left panel) and pepsinogen C (right panel). T47D cells transfected with control or Stat6 siRNAs (500 ng), were harvested at the indicated times for RNA and undergone Quantitative RT-PCR analyses on metallothionein IIA and pepsinogen C mRNA. mRNA levels are expressed relative to levels in control siRNA-transfected cells harvested at 24 h, arbitrarily set as 1. *P ≤ 0.05 versus control. [file 1471-2407-14-10-S9.tiff]

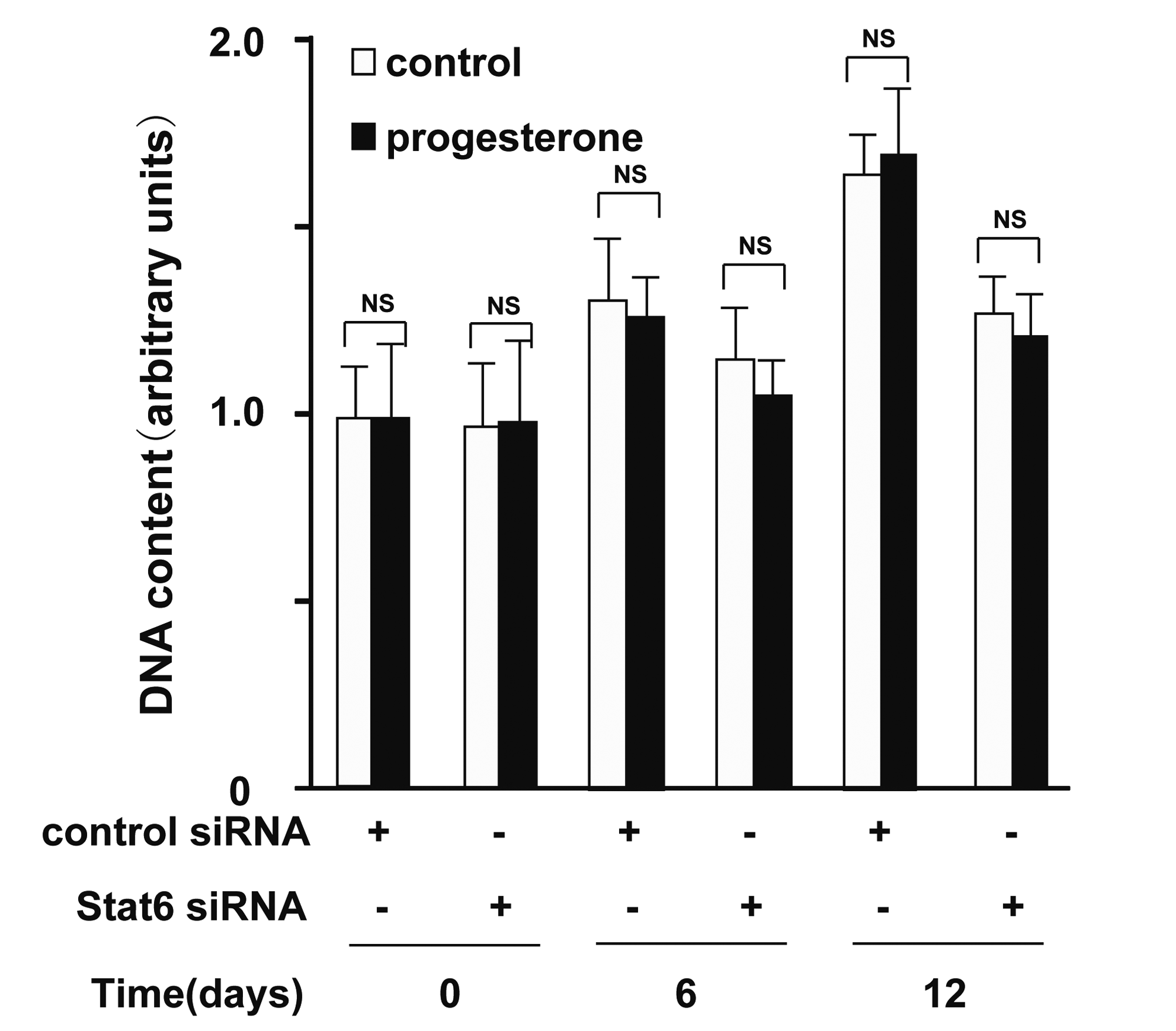

Supplement: Additional file 11: Figure S11 — Knocking-down of Stat6 did not modulate the influence of progesterone on the initial proliferative burst as shown by proliferation assays. T47D cells transfected with control or Stat6 siRNA (500 ng) for indicated time, were harvested and undergone proliferation analyses.*P ≤ 0.05 versus control. [file 1471-2407-14-10-S11.tiff]
